# Dynamic Behavioral and Molecular Changes Induced by Chronic Restraint Stress Exposure in Mice

**DOI:** 10.3390/ijms27010167

**Published:** 2025-12-23

**Authors:** Thomas D. Prevot, Jaime K. Knoch, Dipashree Chatterjee, Sierra Codeluppi-Arrowsmith, Keith A. Misquitta, Corey J. E. Fee, Dwight Newton, Hyunjung Oh, Etienne Sibille, Mounira Banasr

**Affiliations:** 1 Campbell Family Mental Health Research Institute of CAMH, Toronto, ON M5T 1R8, Canada; 2 Department of Psychiatry, University of Toronto, Toronto, ON M5S 1A1, Canada; 3 Department of Pharmacology and Toxicology, University of Toronto, Toronto, ON M5S 1A1, Canada

**Keywords:** chronic stress, behavior, astroglia, GABA neurons, synaptic proteins, co-expression analysis

## Abstract

Chronic stress is a major risk factor contributing to cellular changes in the brain that precipitate the emergence of various behavioral changes, including anxiety and anhedonia—symptoms relevant to mood disorders including major depression—however the sequence and trajectory of early molecular changes is poorly characterized. Using the chronic restraint stress (CRS) model in mice (N = 6–8/sex/group), we assessed the impact of 0, 7, 14, 21, 28, or 35 days of CRS at the behavioral level on the emergence of anxiety-like and anhedonia-like phenotypes. While 7 days of CRS was sufficient to induce anxiety-like behaviors in the PhenoTyper test, anhedonia-like deficits in the sucrose consumption test were only observed after 35 days of CRS. We also investigated the underlying molecular changes in the prefrontal cortex, a limbic brain region highly sensitive to stress, using Western blot and qPCR. We found that protein or RNA levels of several markers known to be implicated in the pathology of depression, and markers of synapses (post synaptic density protein 95 (PSD95), synapsin-1 (SYN1), vesicular glutamate transporter-1 (VGLUT1), and gephyrin (GPHN)); GABAergic inhibitory interneurons (somatostatin (SST), parvalbumin (PV), glutamic acid decarboxylase-67 (GAD67), and vasoactive intestinal peptide (VIP)); and astroglia (glial fibrillary acidic protein (GFAP), glutamate transporter-1 (GLT1), and glutamine synthase (GS)) were gradually reduced by CRS. Interestingly, all three astroglial markers were negatively correlated with anhedonia-like behaviors, while SYN1 and GPHN negatively correlated with anxiety-like behaviors. GLT1, VGLUT1, SYN1, and GAD67 negatively correlated with Z-emotionality scores. Exploratory between-marker correlations and integrative network analyses revealed that CRS effects might be driven by different compartments (synaptic, GABAergic and astroglial) depending on sex. Our study demonstrates that CRS induces dynamic changes that can be observed at the behavioral and molecular levels, and that male and female mice, while exhibiting similar symptoms, may experience different underlying pathologies.

## 1. Introduction

Chronic stress represents a major risk factor for the emergence of major depressive disorder (MDD) [1]. MDD is the most prominent psychiatric illnesses worldwide affecting over 320 million people [2] and causing a significant economic burden [3]. MDD symptoms include anhedonia, low mood, and feelings of worthlessness or helplessness [4], and it is highly comorbid with anxiety disorders [5]. MDD is more prevalent in women than men [6], with women experiencing more severe forms of MDD [7], and being more susceptible to stress [8]. Although psychiatric therapy has greatly improved, increasing the efficacy of current antidepressants is limited [9]. Indeed, only ~60% of patients respond effectively to treatment [10] and others often experience significant side effects and/or relapse [11]. This lack of efficacy is likely due to the fact that current antidepressants mostly target the monoaminergic system, and do not specifically target the primary underlying cellular pathologies of MDD [11], which highlights the need for a better understanding of these pathologies and the trajectory of changes.

In recent years, growing evidence suggests that MDD is characterized by morphological pathologies where brain regions, cells, and cell compartments lose integrity, complexity, and function [12]. Human post-mortem and preclinical studies implicate structural and cellular abnormalities in corticolimbic brain regions, including the prefrontal cortex (PFC) [12], with changes impacting the three main components of cortical cell circuits: excitatory neurons and synapses, GABAergic interneurons, and astroglial cells. Indeed, synaptic loss [13], GABAergic dysfunction [14], and astroglial abnormalities [15] in the PFC have been reported in MDD. Specifically, reduction in density and/or markers of GABAergic neurons (in particular neurons co-expressing somatostatin (SST)) [14] and astroglia [15] are among the most consistent findings reported in MDD, and synaptic loss was found in the PFC of MDD patients [16,17]. Despite their interdependent functional roles, the cellular alterations occurring within the GABAergic, astroglial, and synaptic compartments in MDD are typically studied independently at an advanced stage of illness, preventing the characterization of their emergence.

The development of MDD depends on a complex interplay of genetic, hormonal and environmental variables. One of these variables is psychological stress. Chronic exposure to stress is used in preclinical models to investigate pathological states relevant to MDD [18]. Using these models, studies showed that females are more sensitive to stress exposure [8,19], and tend to develop more severe symptoms than their male counterparts [7,20]. In male and female rodents, chronic stress exposure induces cellular changes similar to those observed in depressed patients [21,22,23]. Several models are used to achieve a chronic stress state, including exposure to unpredictable chronic mild stress (UCMS), chronic restraint stress (CRS), foot shock, social isolation, and social defeat [24]. Here, we opted for the use of CRS since it consistently induces depressive-like behaviors, as well as cellular and morphological changes in neurons and astroglia [23,24,25]. Compared to other stress paradigms, repeated exposure to the same restraint stressor is more suitable for studying time-dependent effects, and as studies have shown, CRS is reliable in inducing synaptic loss in number and/or function contributing to behavioral deficits [19,22,26] in the PFC. GABAergic functions [22,26,27] and astroglial functions [19,28,29] are also altered in the PFC of rodents subjected to chronic stress. Despite evidence that synaptic, GABAergic, and astroglial functions are impacted by chronic stress, the temporal emergence of these changes and contribution to behavioral deficits remains poorly characterized.

Here, we investigated in parallel the cellular alterations induced by CRS across GABAergic, synaptic, and astroglial compartments. We aimed to map their temporal progression, their association with each other and with behavioral deficits. This work is based on the hypothesis that increasing stress duration will cause gradual behavioral and cellular changes. We postulated that the trajectory of the emergence of anxiety-like and anhedonia-like behavioral deficits will be associated with select changes in the expression of markers specific of the synaptic, GABAergic, and astroglial compartments in both male and female mice, incorporating sex as a biological variable. We further used co-expression analyses to explore the coordinated relationship between these markers in order to investigate how exposure to CRS affects the local network (i.e., the network formed by glutamatergic cells, GABAergic interneurons, and astrocytes) in both males and females and explore if the underlying mechanisms involved in the effects of CRS are sex specific.

## 2. Results

### 2.1. Temporal Emergence of Behavioral Outcomes During CRS

**Figure 1:**
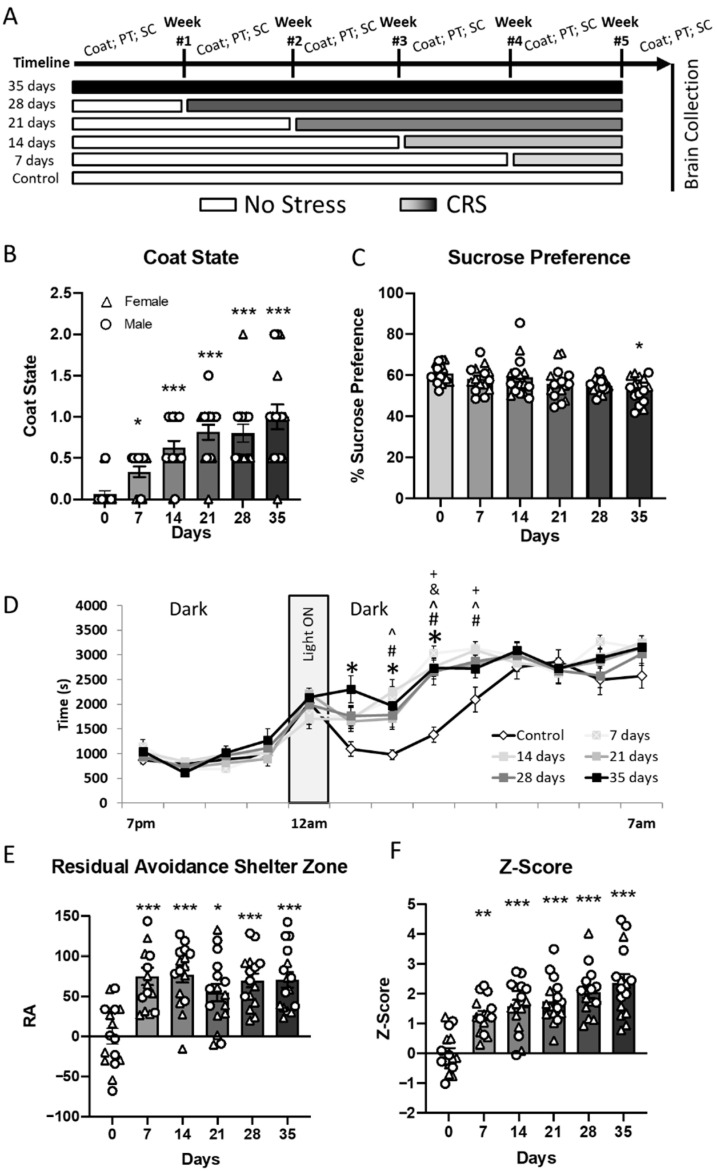
**Chronic restraint stress induces gradual emergence of behavioral changes.** (**A**) Experimental timeline showing different durations of CRS (7 to 35 days) and timing of behavioral assessments: coat state, PhenoTyper (PT), and sucrose consumption (SC). (**B**) Coat state deterioration increased progressively with longer CRS exposure. (**C**) Sucrose preference, an index of anhedonia, significantly decreased only after 35 days of CRS. (**D**) CRS altered daily activity patterns in the PhenoTyper test, with increased shelter zone time after light onset (11 a.m.) in CRS-exposed mice; this effect became more pronounced with increasing CRS duration. (**E**) Residual avoidance (RA) in the shelter zone was significantly elevated in all CRS groups compared to controls, indicating persistent anxiety-like behavior. (**F**) Composite behavioral Z-score showed a significant increase from day 14 onwards, reflecting accumulating behavioral deficits across multiple domains. Data shown as mean ± SEM; individual data points represent male (circles) and female (triangles) mice. * *p* < 0.05, ** *p* < 0.01, *** *p* < 0.001 vs. day 0 or control group; additional symbols denote post hoc comparisons *p* < 0.05 (^ CRS7 vs. CRS0, # CRS14 vs. CRS0, & CRS28 vs. CRS0, + CRS vs. CRS0).

Weight gain, coat state, sucrose preference and performances in the PhenoTyper test were measured weekly (Figure 1A) for all groups. Figure 1 illustrates data obtained from individual groups (between-group analysis) at the end of the experiment i.e., on week 5, after completion of the different stress durations for each group. For within-group analysis, specifically, longitudinal results and weekly monitoring of intermediary timepoints for weight gain and coat state for all groups are presented in Supplementary Figures S1 and S2 and Supplementary Results.

Kruskal Wallis test was used to detect differences in coat state degradation between individual groups subjected to 0, 1, 2, 3, 4 or 5 weeks of CRS (Figure 1B). We identified a significant difference between groups (H = 44.3, *p* < 0.001). Bonferroni/Dunn post hoc test showed significant increase in coat degradation for CRS7 group (*p* < 0.05) and further increasing with greater durations of stress exposure (*p* < 0.001, compared to control group).

Sucrose preference differences were observed only in the animals subjected to CRS for 5 weeks. Indeed, ANOVA of sucrose preference data (Figure 1C) revealed a significant effect of the CRS duration (F_(5,82)_ = 2.9; *p* = 0.015) but no effect of sex or a duration*sex interaction (*p* > 0.05). post hoc Dunnet’s test identified a significant decrease in percent of sucrose consumed versus water in the CRS35 group compared to control (*p* < 0.05).

Figure 1D shows the time spent in the shelter zone (SZ) when all animals were tested on week 5 (weekly assessment of all groups on week 0 to 4 can be found in the Supplementary Figure S3). A repeated measures ANOVA on the time spent in the SZ showed a significant effect of CRS duration (F_(5,984)_ = 4.42; *p* = 0.0012), an effect of sex (F_(1,984)_ = 4.7; *p* = 0.03), and an effect of time (F_(12,984)_ = 146.5; *p* < 0.001) but no CRS duration*sex or CRS duration*sex*time interaction. Dunnet’s test performed on the time spent in the SZ showed no difference in SZ time between 7 p.m. and 11 a.m., and past 4 a.m. (Supplementary Table S2). However, Dunnet’s test showed that animals from the CRS35 group spent more time in the SZ than control between 12 a.m. and 3 a.m. Significant differences compared to the control group were also observed for the CRS28 group between 2 a.m. and 3 a.m., for the CRS21 group at the 2 a.m. timepoint, and for the CRS14 and CRS7 groups between 1 a.m. and 3 a.m. Investigation of the main effect of sex did not identify specific sex-differences. To simplify the visualization of the differences observed between 12 a.m. and 4 a.m., we used the residual avoidance (RA) calculation, as described in Prevot et al. [30], which measures avoidance after a 1 h spotlight challenge. ANOVA performed on the RA-SZ revealed a significant effect of CRS duration (F_(5,82)_ = 10.23; *p* < 0.0001) and sex (F_(1,82)_ = 14.55; *p* < 0.001) with no CRS duration*sex interaction (Figure 1E). post hoc Dunnett’s test identified that animals subjected to CRS displayed greater RA-SZ compared to controls, for all CRS groups (*p* < 0.05–*p* < 0.001). The effect of sex was further characterized, and showed that males from the CRS14, CRS28 and CRS 35 groups exhibited an overall higher RA-SZ than females (Supplement Figure S4). The RA was also calculated based on the time spent in the food zone (FZ) and analysis of the RA-FZ showed similar results to the analysis of RA-SZ (Supplementary Figure S4).

Behavioral data was summarized using a Z-score approach (Figure 1F). ANOVA of the Z-score data showed a significant effect of CRS duration (F_(5,82)_ = 18.2, *p* < 0.001), a significant effect of sex (F_(1,82)_ = 12.9, *p* = 0.0006) and no CRS duration*sex interaction (*p* > 0.5). The effect of sex is explained by an overall higher Z-score in males than females (Supplementary Figure S5). post hoc analyses show that in both males and females there is a significant increase in the Z-score for all CRS duration groups, compared to the control group (*p* < 0.01–*p* < 0.001), starting at week 1 and nominally increasing each subsequent week. These results confirm that CRS exposure induces behavioral changes related to emotionality, and that longer exposure generates more severe deficits.

### 2.2. CRS Exposure Induces Dynamic Changes in Cellular Markers

Western blot analyses for synaptic (SYN1, PSD95, and GPHN), GABAergic (GAD67), and astroglial (GFAP, GS, and GLT1) markers were performed on PFC samples.

**Figure 2:**
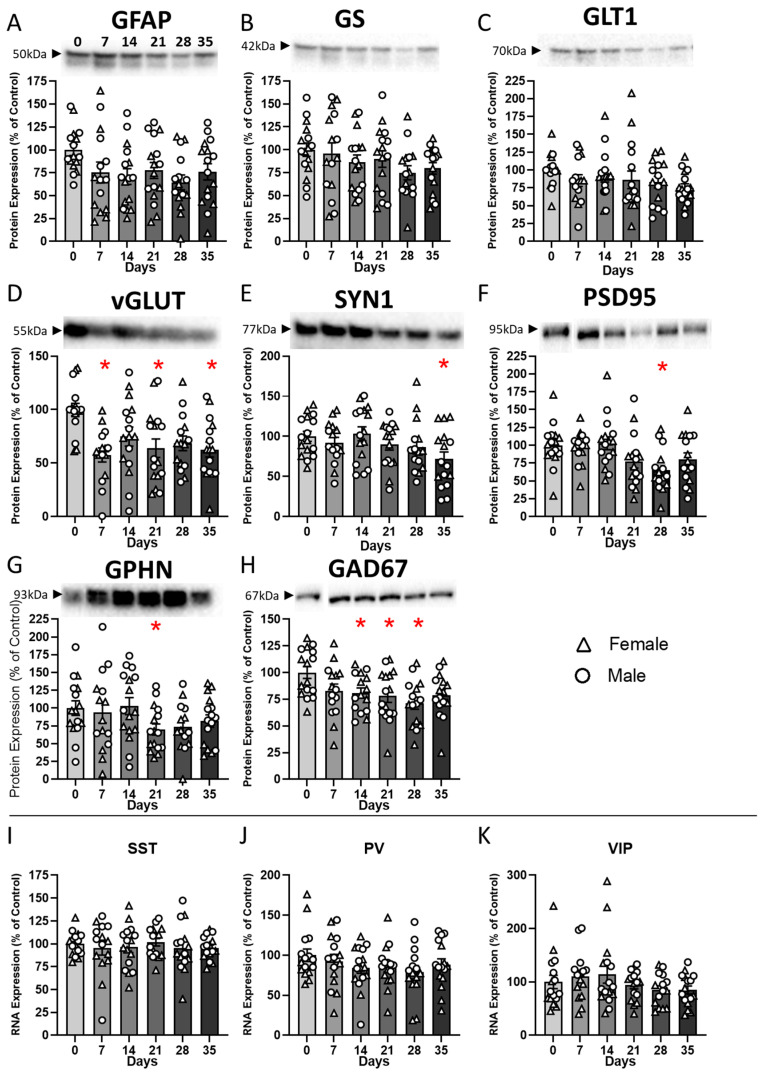
**Chronic restraint stress induces gradual emergence of molecular changes in the PFC.** Relative expression levels (normalized to control) of selected protein (**A**–**H**) and RNA (**I**–**K**) markers were assessed in the prefrontal cortex after 0, 7, 14, 21, 28, or 35 days of CRS. (**A**–**C**) Glial markers: GFAP protein expression decreased significantly by day 28, while GS and GLT1 protein levels were not statistically decreased. (**D**–**F**) Synaptic markers: vGLUT protein levels showed a rapid decrease at 7 days, while SYN1 and PSD95 protein expression were significantly reduced at later time points. (**G**–**H**) GABAergic markers: GPHN protein levels were reduced at 21 days, and GAD67 protein expression was significantly downregulated from day 14 to 28. (**I**–**K**) Expression of interneuron subtype RNAs (SST, PV, and VIP) did not show significant changes across CRS durations. Bars represent mean ± SEM. Individual data points show expression in female (triangles) and male (circles) mice. * *p* < 0.05 vs. control (day 0).

ANOVA of GFAP protein levels revealed a trend in the main effect of CRS duration (F_(5,82)_ = 1.96; *p* = 0.092), a significant effect for sex (F_(1,82)_ = 7.59; *p* < 0.01) and no CRS duration*sex interaction (Figure 2A). Sex main effect is explained by males showing overall higher levels of GFAP protein than females (Supplementary Figure S6A).

ANOVA of GS and GLT1 protein levels revealed no effect of CRS duration (F), sex, or CRS duration*sex interaction (Figure 2B,C).

ANOVA of vGLUT1 protein levels revealed a significant main effect of CRS duration (F_(5,82)_ = 4.1; *p* = 0.002), but no main effect of sex or CRS duration*sex interaction (Figure 2D). Dunnett’s post hoc analysis identified significant decreases in vGLUT1 protein levels in the CRS7, CRS21, and CRS35 groups when compared to controls (*p* < 0.05).

Analysis of SYN1 protein levels showed a significant main effect of CRS duration (F_(5,82)_ = 2.43; *p* = 0.04) and sex (F_(1,82)_ = 12.33; *p* < 0.001) but no CRS duration*sex interaction. We identified a decrease in SYN1 protein levels in the CRS35 group when compared to controls (*p* < 0.05; Figure 2E). Regarding sex effect on SYN1, Dunnett’s post hoc analysis revealed a significant decrease in the CRS35 group in males (Supplementary Figure S6), while no group differences were found in females. Within group comparisons revealed that SYN1 protein levels were significantly higher in CRS7 and CRS35 males compared to females.

Statistical analysis of PSD95 protein levels indicated a significant main effect of CRS duration (F_(5,82)_ = 3.12; *p* < 0.05) but no main effect of sex or CRS duration*sex interaction (Figure 2F), and post hoc analysis revealed a significant decrease in PSD95 protein levels in the CRS28 group when compared to controls (*p* < 0.05).

Regarding GPHN protein expression levels, one sample from the CRS28 group was a significant outlier and was removed from downstream analyses. The ANOVA of GPHN protein levels showed no effect of CRS duration, an effect of sex (F_(1,82)_ = 6.43; *p* = 0.02) and no CRS duration*sex interaction (Figure 2G). The effect of sex was due to an overall lower expression of GPHN protein in females compared to males (Supplementary Figure S6).

Regarding GAD67 protein levels, one sample from the CRS14 group was considered a significant outlier compared to the rest of the group and was removed from subsequent statistical analyses. ANOVA on GAD67 protein levels showed a significant effect of CRS duration (ANOVA: F_(5,81)_ = 2.9; *p* = 0.017), and no effect of sex or CRS duration*sex interaction (Figure 2H), explained by reduced expression levels in the CRS14, CRS21, and CRS28 groups (*p* < 0.05).

Expression levels of SST, PV, and VIP RNA were measured using qPCR. Statistical analysis of SST RNA levels indicated no significant effect of CRS duration, sex or CRS duration*sex interaction (Figure 2I). ANOVA on PV RNA levels identified a significant effect of sex (F_(1,82)_ = 8.6, *p* = 0.004) but no effect of CRS duration or interaction (Figure 2J), explained by a significant decrease in PV RNA in females in the CRS28 and CRS35 groups, compared to males (Supplementary Figure S6). Finally, ANOVA on VIP RNA levels showed no significant effect of CRS duration (Figure 2K) but a significant effect of sex (F_(1,82)_ = 3.8, *p* = 0.05) and a CRS duration*sex interaction (F_(5,82)_ = 2.9, *p* = 0.016). post hoc analyses revealed higher VIP RNA levels in males in the CRS7 and CRS28 groups compared to females (Supplementary Figure S6).

These results confirm that expression changes in synaptic, astroglial and GABAergic markers follow a trajectory often requiring multiple weeks to detect a significant reduction.

### 2.3. Cellular Markers Correlate with Behavioral Outcomes

**Figure 3:**
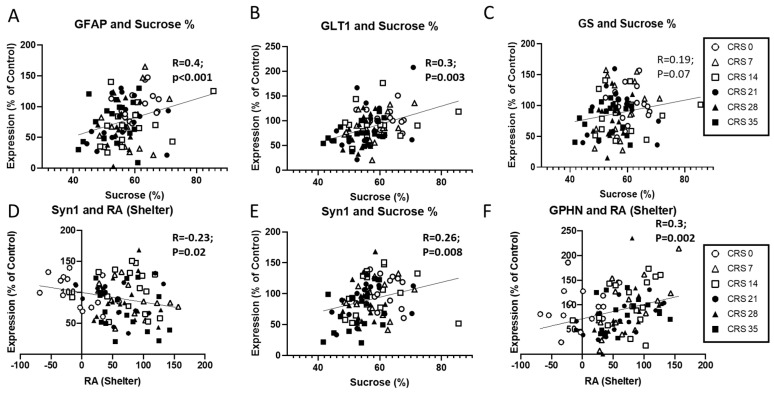
**Correlations between behavioral outcomes and molecular markers in the prefrontal cortex following chronic restraint stress (CRS).** (**A**–**C**) Positive correlations were observed between sucrose preference and the expression of glial markers GFAP (**A**) and GLT1 (**B**), while the association with GS was trending (**C**). (**D**–**F**) Expression of the synaptic marker SYN1 negatively correlated with residual avoidance (RA) in the shelter zone (**D**) and positively with sucrose preference (**E**). GPHN expression was positively associated with shelter RA (**F**). Each symbol represents an individual animal and is coded by CRS duration, as indicated in the legend.

To assess the relationships between the expression levels of the aforementioned markers and behavioral outcomes obtained on Week 5 (i.e., when all groups completed their set duration of CRS exposure; Figure 3 and Supplementary Table S3), we used correlation analyses across groups, independently of sex, and then in males and females separately (see Supplementary Results for details). Using Pearson’s correlation, we found a significant positive correlation between sucrose consumption and GFAP (Figure 3A) and GLT1 (Figure 3B) protein levels. Pearson’s regression analyses also revealed trending correlations between GS protein levels and sucrose consumption (R = 0.19, *p* = 0.07) and RA SZ (R = −0.19, *p* = 0.07). Interestingly, splitting the dataset by sex showed different results. In male mice, all astroglial markers correlated with sucrose consumption (see Supplementary Figure S8 and Supplementary Table S3) while in female mice, only GLT1 protein level correlated with sucrose consumption (R = 0.4, *p* = 0.006). Exclusively in male mice, GFAP and GLT1 protein levels correlated with weight gain and RA-SZ (*p* < 0.04).

Pearson’s regression showed that SYN1 protein levels correlated with RA-SZ and sucrose consumption (Figure 3D,E). Using Spearman’s regression, we found that vGLUT1 protein levels correlated with the coat state score (*ρ* = −0.33, *p* = 0.001). When spitting the dataset by sex these correlations were maintained in males (*p* < 0.05; Supplementary Table S3) but not in females. In addition, PSD95 protein levels positively correlated with sucrose consumption, exclusively in male mice (R = 0.32, *p* = 0.02; Supplementary Table S3). Overall GPHN protein levels were also significantly correlated with RA-SZ (Figure 3F), and coat state score (*ρ* = −0.22, *p* = 0.03). When splitting the data by sex, only the correlation between coat state score and GPHN protein levels remained significant in female mice (*ρ* = −0.5, *p* = 0.017; Supplementary Table S3).

Regarding GABA markers, Spearman’s regression analyses on GAD67 protein expression levels identified negative correlation with coat state (*ρ* = −0.4, *p* < 0.001). Overall VIP RNA level correlated with weight gain (R = 0.24, *p* = 0.018). The only correlation between the coat state scores and GAD67 protein levels remained in female mice after splitting by sex (*ρ* = −0.4, *p* = 0.007; Supplementary Table S3). In male mice, GAD67 protein levels negatively correlated with coat state score (*ρ* = −0.38, *p* = 0.01), and SST RNA expression levels correlated with weight gain (R = 0.35, *p* = 0.01) and RA-SZ (R = −0.33, *p* = 0.026) (see Supplementary Table S3).

These results suggest a relationship between markers and behavioral outcomes. Indeed, GFAP and GLT1—both astroglial markers—correlated with sucrose consumption measures, suggesting a link with anhedonia-like phenotype, while SYN1 levels and GPHN levels –synaptic and GABAergic markers, respectively—correlated with residual avoidance in the shelter, suggesting a link with anxiety-like phenotype.

**Figure 4:**
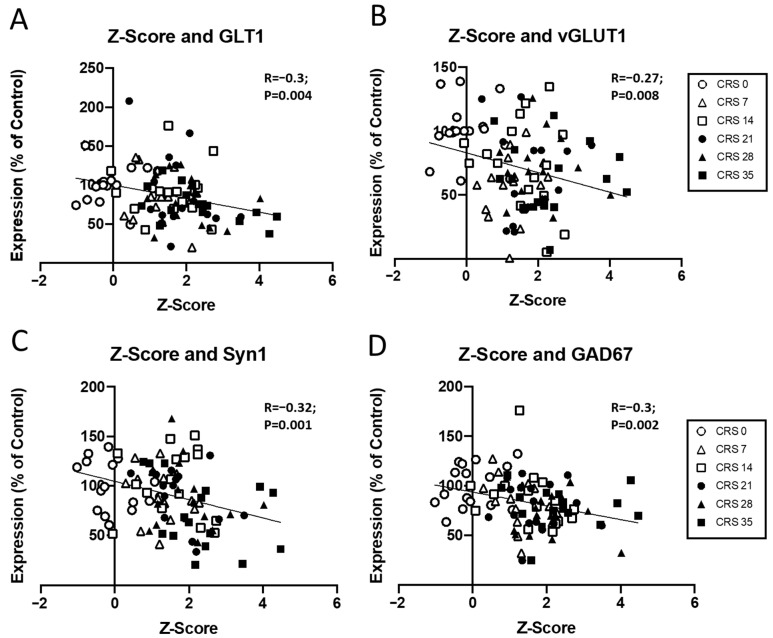
**Correlation between molecular changes and Z-scores.** Scatter plots showing the relationship between Z-score and the expression levels of (**A**) GLT1, (**B**) vGLUT1, (**C**) Syn1, and (**D**) GAD67. Data are presented as the percentage of control expression, with individual data points representing different groups (CRS 0, CRS 7, CRS 14, CRS 21, CRS 28, and CRS 35). The correlation coefficients (R) and *p*-values are shown for each plot, indicating statistically significant inverse correlations between Z-score and expression for all genes tested. Each symbol represents an individual animal and is coded by CRS duration, as indicated in the legend.

Finally, possible correlations between all markers and Z-emotionality scores were investigated (Figure 4; Supplementary Table S3). Z-score correlated with GLT1, vGLUT1, SYN1, GAD67 protein levels (Figure 4) and was trending towards significance with PSD95 (R = −0.18, *p* = 0.08). After splitting by sex, different profiles emerged for males and females (Supplementary Figures S7–S9). In males, significant correlation was found with GLT1 (R = −0.4, *p* = 0.0004), GFAP (R = −0.29, *p* = 0.04), SYN1 (R = −0.36, *p* = 0.001), GAD67 (R = −0.3, *p* = 0.01) protein levels and was trending towards significance with vGLUT1 (R = −0.26, *p* = 0.07) and PSD95 protein expression (R = −0.18, *p* = 0.06). In females, only vGLUT1 protein levels was significantly correlated with the Z-score (R = −0.4, *p* = 0.005), while GPHN protein expression was trending towards significance (R = −0.25, *p* = 0.08). These results suggest a contribution of markers such as GLT1, vGLUT1, Syn1 and GAD67 in the emergence of emotionality due to CRS exposure.

### 2.4. Progressive Sex-Specific Shift of PCA Loadings with Increased Durations of CRS

**Figure 5:**
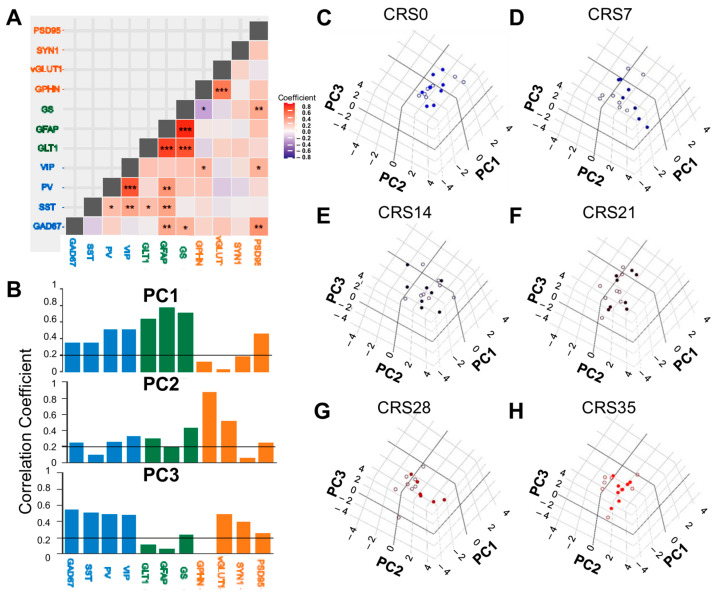
**Marker expression analyzed using pairwise correlations and principal component analysis.** (**A**) Correlation matrix showing pairwise relationships among markers; correlations were performed on data obtained across both sexes and all stress groups. Asterisks represent statistical significance of r values calculated using Pearson’s correlation and corrected using Benjamini-Hochberg FDR. * denotes q < 0.05; ** denotes q < 0.01, *** denotes q < 0.001. In (**A**,**B**), blue denotes GABAergic markers, green denotes astroglial markers, and orange denotes synaptic markers. (**B**) Histogram representing loadings of individual markers on the first three principal components (PC1–PC3), illustrating the relative contribution of each variable to variance capture by each PC. The horizontal black line marks the critical r value of statistical significance for 55 unique pairwise comparisons (α = 0.05). (**C**–**H**) 3-D PCA plots displaying each mouse projected onto PC1–PC3 for each group. The axes have been oriented to visually highlight the progressive differential migration between the sex groups through the weeks of CRS exposure. In (**C**–**H**), each circle represents a mouse, filled circles denote males and open circles denote females.

The relationship between the expression levels of the different markers with each other was then analyzed (Figure 5A, Supplementary Figure S10 and Supplementary Table S4). Pairwise Pearson’s correlation analysis was performed and showed that most markers were positively correlated with each other (Figure 5A), with the protein levels of astroglial markers GLT1 and GFAP (R = 0.545, *p* < 0.0001), as well as GLT1 and GS (R = 0.491, *p* < 0.0001), and GFAP and GS (R = 0.627, *p* < 0.0001) being particularly strongly correlated with each other. GS and GPHN were the only proteins that were significantly negatively correlated with each other (R = −0.23, *p* = 0.027; Figure 5A). Interestingly, none of the astroglial markers correlated with the pre-synaptic markers (Syn1 and vGLUT1). However, GS correlated with the post synaptic markers PSD95 (R = 0.27, *p* = 0.009) and GPHN (R = −0.23, *p* = 0.02). GS also correlated with GAD67 protein levels (R = 0.24, *p* = 0.017), and was trending with PV (r = 0.17, *p* = 0.09) and VIP (R = 0.19, *p* = 0.057) RNA levels. GLT1 protein levels correlated with SST RNA levels (R = 0.21, *p* = 0.03) and was trending with VIP RNA expression (R = 0.2, *p* = 0.06).

Exploratory analyses using principal component analysis (PCA) was then performed to investigate the broad patterns of marker expression across durations of stress. All protein and RNA level datasets of all groups regardless of sex were included. PCA showed that the first five PCs accounted for about 75% of the total variance in the data with the first three PCs explaining 24.5%, 14.6%, and 14.1% respectively (Supplementary Table S5). Analysis of Pearson’s correlation coefficients of each marker with PC1, PC2, and PC3, showed that PC1 primarily loaded the astroglial and GABAergic markers, while PC2 loaded the majority of the synaptic markers, and PC3 loaded a portion of the GABAergic and synaptic markers (Figure 5B). Due to the relatively equal weighting of PC2 and PC3 on the variance explained, we plotted three-dimensional scatterplots in order to visualize the data (Figure 5C–H; Supplementary Figure S11). The PCA confirmed that the synaptic markers SYN1, PSD95, VGLUT1, and GPHN clustered together; the GABAergic markers SST, PV, GAD67 and VIP clustered together, and so did the astroglial marker GFAP, GLT1, and GS. This data driven-clustering corroborates the functional and compartment-specificity of these markers. We then performed statistical analyses on the PCs, to determine if stress or sex influence the variation of specific clusters of markers loaded on the PCs. We found a significant main effect of stress on PC1 (F_(5,82)_ = 3.225, *p* < 0.05), a trending main effect of sex on PC2 (F_(1,82)_ = 3.322, *p* = 0.072) and both main effects on PC3 (F_(5,82)_ = 2.260, *p* = 0.05; F_(1,82)_ = 13.353, *p* = 0.0005, respectively) but no interaction. Correspondingly, it appears that there is a sex-specific progressive shift from greater loadings with PC1 and PC2 in the CRS0 group to PC1 and PC3 with increasing durations of stress, distinguishing males and females after 28 days of CRS (Figure 5C–H). These results implied some sex-specific changes in the importance of the GABAergic, synaptic and astroglial compartments in addition to the variability due to chronic stress, and suggested that follow-up analysis should explore potential sex differences.

### 2.5. Astroglial, GABAergic, and Synaptic Network Alterations Induced by CRS Are Sex-Dependent

**Figure 6:**
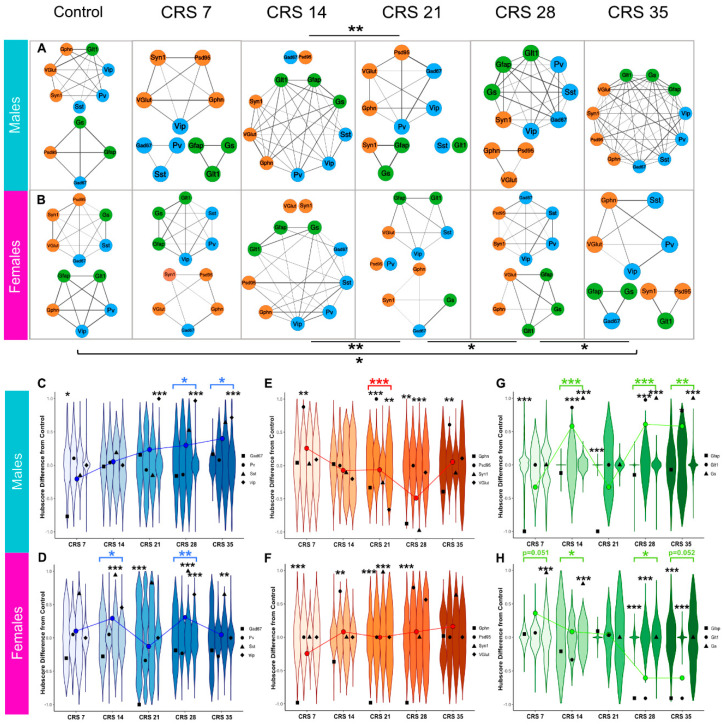
**Marker coexpression network analysis and hubscore changes of individual markers and GABAergic, synaptic, and astroglial compartments over the course of CRS.** Co-expression analysis network diagrams for the male (**A**) and female mice (**B**) subjected to various durations of CRS (0, 7, 14, 21, 28, 35 days). Lines or “edges” show the strength of coexpression between markers, where thickness of lines is proportional to correlation of expression. Markers without edges strong enough to form a module are shown separately without edges. Asterisks represent statistical significance after Benjamini-Hochberg FDR correction, where * denotes q < 0.05; ** denotes q < 0.01. Blue denotes GABAergic nodes, green denotes astroglial nodes, and orange denotes synaptic nodes. (**C**–**H**). Violins show the null distribution of permuted hubscore differences, and black points represent the observed hubscore for each marker. The overlayed line plots the trajectory of average hubscore for entire compartment, and the asterisks of the same color represent a significant change in hubscore for the entire compartment compared to CRS0, as computed by Fisher’s *p* meta-analysis. (**C**–**E**) Male marker hubscore changes compared to CRS0 separated by compartment (blue for GABAergic (**C**), orange for synaptic (**D**), and green for astroglial (**E**)). (**F**–**H**) Female marker hubscore changes compared to CRS0 separated by compartment (blue for GABAergic (**F**), orange for synaptic (**G**), and green for astroglial (**H**)). * *p* < 0.05; ** *p* < 0.01; *** *p* < 0.001.

To investigate further the effect of stress duration on the expression of the GABAergic, astroglial, and synaptic markers in a sex-dependent manner, network co-expression analysis was performed. We found a notably different reorganization of markers in response to stress between males (Figure 6A) and females (Figure 6B). While both sexes showed significant changes in the makeup of networks across stress durations, only the females had a significantly different network reorganization at CRS35 when compared to the CRS0 group (*p* = 0.0078, q = 0.014), where the network becomes more fragmented after CRS. Females also showed significant network changes between each week from CRS14 onward, with CRS14 to CRS21 (*p* = 0.021), CRS21 to CRS28 (*p* = 0.0054, q = 0.0096), and CRS28 to CRS35 (*p* = 0.01, q = 0.018) (Figure 6B). Males showed an early reorganization in response to stress between CRS14 and CRS21 (*p* = 0.013) and appear to undergo further reorganization resulting in a highly organized single network including all markers at CRS35 (Figure 6A); however, this effect was a trend (*p* = 0.099).

Although the network analysis yielded interesting results, the method considers each marker “independently” regardless of its function or the cell type that expresses it. To address this limitation and push the analysis further, we classified groups of markers into three compartments: GABAergic (GAD67, SST, PV, and VIP), synaptic (VGlut1, PSD95, Syn1, and Gephrin) and astroglia (GLT1, GFAP, and GS) and performed hubscore analysis in order to determine if the importance of each compartment in the network changed following CRS exposure. Hubscore analysis was performed to evaluate CRS effects on the relative importance of each marker in the network (Supplementary Tables S6 and S7). Regarding the GABAergic markers, in males, the CRS7 group showed a decrease in the hubscore of PV as compared to CRS0 (*p* = 0.015) (Figure 6C), followed by no change in CRS14. GAD67 hubscore increased at CRS21 (*p* < 0.0001), 28 (*p* < 0.0001) and 35 (*p* < 0.0001). In the females, (Figure 6F) SST hubscore increased at CRS14 (*p* < 0.0001), 28 (*p* < 0.0001), and 35 (*p* = 0.009). Females also showed a decrease in the hubscore of PV in the CRS21 group (*p* < 0.0001).

Regarding the synaptic markers, in males (Figure 6D) CRS7 showed an increase in PSD95 hubscore compared to CRS0 (*p* = 0.0126), but no changes in CRS14. The CRS21 group displayed an increase in PSD95 hubscore (*p* < 0.0001) and a decrease in SYN1 hubscore (*p* = 0.0019), while CRS28 showed decreases of both GPHN (*p* = 0.003) and VGLUT1 (*p* < 0.0001) hubscores, and PSD95 hubscore was increased in the CRS35 males (*p* = 0.0032). The females (Figure 6G) showed increased PSD95 hubscore at CRS14 (*p* = 0.0136), as well as decreased hubscore of GPHN at CRS7 (*p* < 0.0001), CRS21 (*p* < 0.0001), and CRS28 (*p* = 0.0002). There was also an increase in VGLUT1 hubscore CRS21 females (*p* = 0.0008).

Regarding the astroglial markers, in males (Figure 6E), the GLT1 hubscore decreased in CRS7 (*p* < 0.0001) and CRS21 (*p* < 0.0001) groups compared to CRS0, while GFAP and GS had increased hubscores in the CRS14 (*p* < 0.0001; *p* < 0.0001), CRS28 (*p* < 0.0001; *p* < 0.0001), and CRS35 (*p* = 0.00277; *p* < 0.0001) groups, respectively. Oppositely, an increase in the GS hubscore was found in the CRS7 (*p* < 0.0001) and CRS14 (*p* < 0.0001) female groups, with decreased GLT1 and GFAP in the CRS28 (*p* < 0.0001; *p* < 0.0001) and CRS35 (*p* < 0.0001; *p* = 0.0025) females, respectively (Figure 6F).

Finally, using Stouffer’s meta *p* analysis we investigated the effect of CRS on the GABAergic, synaptic, and astroglial compartments as a whole using average compartment hubscores. Concerning the GABAergic compartment, in males, (Figure 6C) hubscores increased in CRS28 (*p* = 0.0158) and CRS35 (*p* = 0.0382) groups. In the females, GABAergic hubscores increased in the CRS14 (*p* = 0.0139) and CRS28 (*p* = 0.0034) groups (Figure 6F). Regarding the synaptic compartment, the males showed a decrease in hubscore in the CRS21 group (*p* = 0.0004) (Figure 6D) and there were no changes in females (Figure 6H). In the astroglial compartment, an increase in hubscore in males (Figure 6E) was found in the CRS14 (*p* < 0.0001), CRS28 (*p* < 0.0001), and CRS35 (*p* = 0.0074) groups. Females showed a marginal increase in hubscore in the CRS7 group (*p* = 0.051) and the CRS14 group (*p* = 0.016) compared to CRS0. The CRS28 group showed a reduction (*p* = 0.012) and the CRS35 showed a marginal reduction (*p* = 0.052) compared to CRS0 (Figure 6H).

## 3. Discussion

In this study, we examined the impact of various durations of CRS exposure in mice on anxiety- and anhedonia-like behavior as well as on the expression of key functional markers of the GABAergic, synaptic, and astroglial compartments. We also investigated the link between these markers and behavior, and how CRS altered the relationship between the markers, individually and across compartments. We demonstrated that anxiety-like behaviors are the first behavioral response to emerge during CRS exposure, followed by anhedonia-like behaviors with longer exposure. At the molecular level, we demonstrated that CRS induces a progressive reduction in markers of function and structure of astrocytes, GABAergic interneurons, and synapses. Using correlation, principal component and network analyses, we showed that the impact of CRS is dependent on its duration, and that, while showing similar behavioral and molecular changes, male and female mice have distinct responses to CRS when considering network reorganization as a whole, or the role of each compartment within the network.

We confirmed that chronic stress exposure induces behavioral changes as early as 1 week after initiation of the CRS paradigm. Using the PhenoTyper test of anxiety [25], we showed that anxiety-like behaviors are present from week 1 and onward [22,25,28] further confirming the validity of this test in detecting anxiety-like behaviors in a repeatable fashion. Other studies also showed that CRS induces anxiety-like behaviors in rodents using other behavioral assays [31,32] or designs (3 h/day for 14 days in [32]; and 6 h/day for 28 days in [31]). We also found anhedonia-like behaviors emerged more gradually, detectable only after 35 days of CRS, in accordance with other studies [33]. This trajectory highlights how critical the duration of chronic stress is on specific behavioral outcomes. One could postulate that there is a biphasic mechanism in place that represents an adaptive response to chronic stress, marked by anxiety-like behaviors at first which progresses into a pathological response combining anxiety-like and anhedonia-like behaviors. These phases may rely on specific underlying cellular mechanisms.

We also investigated the trajectory of CRS effects on a small selection of markers known for their roles in the glutamatergic and GABAergic tripartite synapse in the PFC that are known to have reduced expression in post mortem brains of MDD patients [34]. We found that vGLUT1 was the first and only marker reduced after 1 week of CRS. This is consistent with previous studies showing that reduced levels of vGLUT1 in the PFC increased susceptibility to chronic stress [35] and facilitated the emergence of anxio-depressive-like behaviors and neuroendocrine responses in rodents [36]. With longer CRS exposure (14–21 days), GAD67 and GPHN levels were also reduced, which is similar to reductions described in other chronic stress paradigms [12,27,37], and in depressed patients [38]. At later stages of CRS exposure (28–35 days), vGLUT1 protein expression was still low and accompanied by reduced GFAP, SYN1, and PSD95 protein expression. These results are consistent with morphological studies reporting decreased astroglial complexity [28,39] and synaptic loss [40,41,42] in chronic stress animals and in MDD brains [16,43]. While chronic stress-induced GFAP reductions are well characterized [44,45,46,47], in this trajectory study, decreased GFAP protein expression was found early on during CRS exposure but was not significant because of high inter-individual variability. Importantly, females displayed GFAP reductions throughout CRS exposure. Although the marginal reductions in the investigated astroglial markers’ expression did not reach significance in this study, reduced GS and GLT1 protein expression have been described in other stress models [47,48,49].

We found decreases in SYN1, PSD95 and GPHN protein expression after 35 days for SYN1 and 28 days of CRS for PSD95 and GPHN proteins, confirming previous findings of reduced SYN1 protein expression in the PFC or hippocampus after chronic stress [50,51], and PSD95 reductions following chronic corticosterone administration [52]. Similar decreases in PSD95 levels and synaptic loss were reported in the PFC of MDD patients [17,53].

GAD67 reductions were found after 2 weeks of CRS, confirming previous studies using other chronic stress paradigms [12,27,37]and brains from MDD patients [38]. Interestingly, this reduction in GAD67 protein happened only 2 weeks after CRS induction, while in MDD patients, reduced GABA levels were reported in postmortem tissue. This suggests that GAD67 reduction, and by extension reduction in GABA levels could happen in the earliest stages of MDD. However, here, we did not find significant reductions in SST, PV, or VIP RNA levels, which is inconsistent with previous findings. While mixed results have been reported regarding PV expression changes in the PFC following stress [12,54], SST levels are consistently found reduced in postmortem brain of MDD patients [14,55] and in chronic stress animals [56]. Reductions in VIP levels have also been reported following chronic stress [12].

Previous studies have shown an association between astroglial dysfunction and depressive-like behaviors [15,30,47,57,58]. Here, we demonstrated that all three astroglial markers investigated (GFAP, GS, and GLT1) positively correlated with sucrose consumption. This suggests that animals with reduced astroglial function exhibited anhedonia-like behaviors and confirms recent findings showing that ablation or enhancement of activity of GFAP-astroglia induces anhedonia [21]. The strong association between PFC astroglia and anhedonia-like behaviors suggests that the astroglial dysfunction displayed after longer durations of CRS may be a tipping point leading to the development of anhedonia-like deficits. Concerning synaptic dysfunction, synaptic loss has been reported following acute, sub-chronic, and chronic stress [42,59]. Here, we found that the synaptic marker SYN1 strongly negatively correlated with anxiety- and anhedonia-like behavior, suggesting that unlike astroglial dysfunction, synaptic impairment or loss is involved in both behaviors. Additionally, GABAergic markers exclusively correlated with anxiety-like behaviors. The link between the GABAergic system and anxiety-like behaviors is well-supported by a plethora of studies investigating GABAergic anxiolytic agents and demonstrating anxiety-like deficits in rodent models with impaired GABAergic function [60,61]. This is also in accordance with human findings of reduced GABAergic transmission [14] or SST and GAD67 reductions in brains of MDD patients with anxiety symptoms or with comorbid anxiety disorders [14,38,62]. Altogether our data suggest that GABAergic, synaptic, and astroglial dysfunction may underlie specific behavioral deficits associated with chronic stress.

Sex differences have been reported at the cellular and behavioral levels following chronic stress [63,64]. Throughout this study, analyses of behavior, cellular markers, and their correlation consistently revealed stronger effects and associations in males, and slight expression differences between males and females. This latter observation was further highlighted when we conducted PCA, which ultimately revealed an interesting temporal pattern that suggested a sexual dichotomy in the trajectory of marker expression with increased CRS duration. Using marker co-expression network analysis [65], we found that males and females underwent different network reorganizations following CRS. Generally, there was a common response to the shorter CRS durations, where both sexes showed a loss of connectivity in the marker co-expression network. However, with longer CRS exposure, the co-expression network became more connected in males, but more disconnected in females, suggesting sex-dependent changes in CRS-induced network connectivity. Other studies have found contrasting network reorganization when comparing the responses of both sexes to chronic stress and MDD [7,66]. Indeed, Labonté et al., 2017, found that there was very little overlap in the males’ and females’ transcriptomic networks in both MDD patients and chronic stress mice [7]. Altogether, the dissimilar patterns of network reorganization between sexes in CRS or MDD are not unexpected, although it further complicates our understanding of the underlying pathological mechanisms of chronic stress and MDD.

We used coexpression network analysis to explore if CRS affected the GABAergic, synaptic, and astroglial compartments differently. To test this hypothesis, we used Kleinberg’s centrality, an elegant measure that encompasses the centrality, degree, and connection strength of each node in a network. This approach allows us to essentially measure a node’s importance in the network [67]. We found that CRS increased GABAergic nodes’ importance in the network, but this effect relied on different markers: GAD67 in males and SST in females. These findings are consistent with reports of GABAergic impairment in MDD and chronic stress, and greater SST cell vulnerability in females [14,66,68]. Although we did not find overall changes in the role of the synaptic compartment in the network, the importance of PSD95 steadily increased in both sexes across CRS durations, while GPHN’s importance decreased. These opposite effects support the previously reported shift in the excitatory-inhibitory balance toward greater excitatory and lower inhibitory activity with chronic stress [69,70] and MDD [71]. We found that the importance of the astroglial compartment in the network strengthened in males and lessened in females with increased CRS duration. These results suggest that CRS-induced expression changes in the astroglial compartment may affect the coexpression network in a sex-dependent manner, by contributing to the disruption within tripartite synapse network in males and by disconnecting from the synaptic and GABAergic compartments of the network in females. Altogether, this finding supports the growing collection of evidence that chronic stress may have sexually dimorphic effects on astroglial cells in the cortex [72].

This study also presents some limitations. To start with, we did not monitor the estrous cycle of the female mice. We also only focused on the PFC, one of the many other brain regions that are impacted by chronic stress and MDD, e.g., the hippocampus or the amygdala. It could be of interest to perform the same study in the hippocampus and the amygdala in order to investigate the gradual impact of CRS in these brain regions and complement our network analyses in a more integrative fashion. Also, we used CRS as a model to induce behavioral and molecular changes in mice, while other studies use chronic unpredictable mild stress. We purposely chose the CRS model to be able to detect differences between stress durations, which would have been more difficult to achieve with the relatively milder stress effect of UCMS across timepoints. Another limitation is that we restricted our investigation to select markers of 3–4 compartments (GABAergic, pre- or post- synaptic, and astroglial compartments), and performed exploratory correlation analyses across groups, as well as PCA and network analyses despite the small dataset. Follow-up studies could investigate additional markers or perform RNA-sequencing in each cell type to determine gene-expression changes caused by CRS duration, using nucleus sequencing for example. However, sequencing studies rely on RNA, and not protein level. For this first investigation, we wanted to use protein measurements when possible in order to capture changes in the synaptic compartments. Finally, our analyses were performed on samples from bulk PFC and must be integrated with more of the recent literature using single-cell or single-cell type RNAseq approaches identifying gene expression changes in GABAergic neurons [70], glutamatergic neurons [73], or astroglial cells [72] in animals subjected to chronic stress.

To conclude, we show that CRS induces behavioral and molecular changes in a time-dependent, sex-dependent, and compartment-dependent manner. We highlight that shorter durations of CRS exposure may be models of an acute stress response, while longer CRS exposure may induce more “pathologically relevant” behavioral and molecular changes, aligned with what can be observed in MDD. We also report that while inducing apparently similar symptom expression and cellular changes, CRS effects may rely on distinct underlying network organizations that may explain the underlying sexual heterogeneity in incidence and severity associated with chronic stress and depression.

## 4. Materials and Methods

### 4.1. Animals

Eight-week-old C57BL/6 male and female mice from Jackson Laboratories (Stock No: 000664; Bar Harbor, ME, USA) were used. After a 1-week habituation period to the facility under a regular 12 h light/dark cycle and ad libitum access to food and water, 96 mice were assigned to either the control group or one of the CRS groups. CRS-exposed mice were subjected to 7, 14, 21, 28, or 35 days of CRS with n = 8/group/sex at study initiation. However one male in the CRS7 group and one male in the CRS28 group were removed from the study after 2 weeks of CRS due to health concerns. Upon veterinarian assessment, the animals were euthanized and not included in the analyses. During the CRS paradigm, mice were single-housed with minimum nesting material (only Nestlet square (Ancare^®^), Bellmore, NY, USA). Mice from the control group were also single housed to allow for accurate measurement of sucrose consumption and given nesting material (Nestlet square (Ancare^®^) and nesting cup (The Andersons Bed-r’Nest, Quakertown, PA, USA). CRS and control mice were housed in a separate room to avoid any disruption. Control mice were handled daily for 3 days before the start of experiments to reduce stress from interaction with the experimenter [74]. All experiments were conducted in line with guidelines provided by the Canadian Animal Care Committee and animal use protocol was approved by the animal care committee of the Centre for Addiction and Mental Health (CAMH)-Certificate# 0038 (AUP#987).

### 4.2. Chronic Restraint Stress (CRS)

The CRS protocol consists of placing the mice in a 50 mL Falcon^®^ Tube, with a hole at the bottom and on the cap to allow air flow [75], for 1 h, twice daily (between 9 a.m. and −6 p.m., minimum 2 h apart between daily restraint stress sessions), every day for 7, 14, 21, 28, or 35 consecutive days.

### 4.3. Coat State and Body Weight Assessments

Coat state was assessed following the method described by Yalcin et al. [76]. Seven body parts were assessed for overall appearance of the coat (head, neck, dorsal coat, ventral coat, tail, forepaws, and hind paws). Each body part was given a score of 0 for a well-groomed coat, 0.5 as an in-between, or 1 for a deteriorated coat. Weight recorded at the beginning every week at 9–10 a.m. and weight gain was calculated using the weight of each mouse on week 0 as reference and expressed as percent change. Coat state and body weight were measured weekly.

### 4.4. Sucrose Consumption Test

Sucrose consumption test was performed as in [21,22,28] weekly, at least 24 h after the PhenoTyper test. Mice (n = 7–8/group/sex) underwent habituation to a 1% sucrose solution during 48 h for the first exposure and 24 h for subsequent weeks. After each habituation, mice were then fluid-deprived for a 16 h overnight period (~6 p.m. to 10 a.m.). On the subsequent morning, the sucrose solution was returned to the mice for a 1 h period after which consumption was recorded. This same protocol was repeated with drinking water for water consumption to be used for comparison. Each week, sucrose preference was calculated and expressed as percent of total fluid consumed (sucrose + water), and used as a measure of anhedonia-like behavior.

### 4.5. PhenoTyper Test

The PhenoTyper^®^ test of anxiety was used on a weekly basis to assess anxiety-like behavior as per the protocol described in Prevot et al. [25]. Briefly, every week, mice (n = 7–8/group/sex) were placed in the PhenoTyper^®^ boxes (Noldus, Leesburg, VA, USA) overnight, in which their overall activity was monitored from 7 a.m. to 7 p.m. by an infrared camera, mounted in the ceiling of the box, and linked to Ethovision^®^ XT 16 software (Noldus). At 11 p.m., and for 1 h, a light challenge was applied above the food zone, and time spent by the animal in the food zone (FZ) or the shelter zone (SZ) was monitored. Using the time spent in the FZ and SZ during the 5 h after the light challenge, two residual avoidance (RA) scores were calculated based on the formula previously described in details in Prevot et al. [25]. This RA is a percentage avoidance calculated for each animal that considers the difference between the animal’s response during the light challenge and the sum of time spent avoiding the lit zone for the following 5 h normalized in reference to the control group. Thus, control animals have an RA score of 0 while animals subjected to CRS have consistently shown a positive RA score [25]. An increase in RA score is used as a proxy of anxiety-like behavior.

### 4.6. Sample Collection and Preparation

To focus on the long-term molecular changes caused by CRS and not the immediate effect of restraint stress, animals were euthanized at the same time of the day (9–11 a.m.) 18 h after the last stressor via instant live decapitation. Brains were collected, and the PFC was dissected and then frozen on dry ice. Using a Qiagen Allprep RNA/protein Kit (#80404, Markham, ON, Canada), RNA and proteins were extracted from the PFC samples, as per the provider’s guidelines. RNA concentration was measured using a Nanodrop (Implen NanoPhotometer^®^ P360, Atascadero, CA, USA) apparatus and converted into complementary DNA (cDNA) using a SuperScript VILO cDNA Synthesis Kit (ThermoFisher, Waltham, MA, USA, Cat#: 11754050). Protein levels were quantified using a Pierce BCA (Bicinchoninic Acid) Protein Assay Kit (ThermoFisher, Waltham, MA, USA, Cat #: 23250). cDNA and protein samples were stored at −80 °C.

### 4.7. Western Blot and qPCR

Protocols used were performed as per standard in the field [77], with primers described in Supplementary Methods. In brief, samples (n = 6–8/group/sex) were processed by Western blot, carried on BioRad Criterion TGX Stain-Free Precast gels (4–20%), and transferred onto nitrocellulose membrane. Primary antibody incubation included the following targets: postsynaptic density protein-95 (PSD95), synapsin-1 (SYN1), gephyrin (GPHN), vesicular glutamate transporter-1 (VGLUT1), glutamate transporter-1 (GLT1), glial fibrillary acidic protein (GFAP), glutamine synthase (GS), and glutamic acid decarboxylase-67 (GAD67). Respective secondary antibodies (see Supplementary Table S1 for details) were then incubated followed by exposure to Enhanced Chemiluminescence (ECL), and imaged and quantified using a molecular imager (ChemiDoc XRS, BioRad, Mississauga, ON, Canada) and ImageLab™ 6.1 software (BioRad). Results were normalized to total protein and expressed as a percentage of the control group. For measurements of GAD67, one sample was not successfully measured in the CRS14 group, bringing the total N to 15. In the GPHN measurements, similar issues were noted in the CRS28 group bringing the number of samples analyzed down to 14 for this marker.

cDNA samples (n = 7–8/group/sex) were processed by qPCR to amplify cDNA of somatostatin (SST), parvalbumin (PV), and vasopressin (VIP), on a Mastercycler real-time PCR machine (Eppendorf, Hamburg, Germany). All the primers used were purchased from Integrated DNA Technologies (IDT; Coralville, IA, USA) and specific details can be found in the Supplementary Methods. Results were normalized to three validated internal controls (actin, glyceraldehyde 3-phosphate dehydrogenase (GAPDH), and cyclophilin G) and calculated as the geometric mean of threshold cycles. The results are expressed as a percentage of the control group.

### 4.8. Statistical Analysis

An a priori power analysis was conducted to determine the trajectory of changes induced by chronic restraint stress (CRS). Based on an estimated power of 0.45–0.5, the required sample size ranged from 86 to 100 animals in total. This corresponded to approximately 16 animals per group, which exceeds the standard sample sizes commonly reported in the literature for comparable behavioral and molecular analyses in rodents. Although sex differences were not the primary focus of this study, we included a sufficient number of animals to explore sex effects to some extent. Achieving full statistical power to robustly assess sex-specific effects would have required more than 300 mice, which was deemed disproportionate and not ethically justifiable. Statistical analysis of behavioral outcomes and expression levels was conducted using StatView 5.0 software (Statistical Analysis System Institute Inc., Cary, NC, USA). Normal distribution of the data was assessed with Shapiro Wilk test prior to statistical analysis. Data following a normal distribution were analyzed using ANOVA with stress, time and sex as factors. Dunnet’s post hoc tests were conducted when main effects of stress or sex were significant, in order to compare the impact of the different durations of CRS to the control group, or to compare males and females. Data with a non-normal distribution were analyzed using the non-parametric Kruskal Wallis test, followed by Bonferroni/Dunn post hoc tests. Behavioral measurements, including coat state score, weight gain, residual avoidance in the shelter (SZ RA) and the food zone (FZ RA), as well as sucrose consumption were used to generate an overall Z-score, as per the method described in Guilloux et al. [78]. Z-scores were calculated in a sex-dependent manner, using the control mice of each sex as the reference group.

Covariance between behavioral measures (weight gain, FZ RA, SZ RA, sucrose consumption and Z-scores) and molecular markers, as well as between markers (Supplementary Materials), was assessed by Pearson’s correlation and linear regression. Because of the ordinal distribution of the coat state scores, Spearman’s rank regression analyses were used for correlating markers with coat state scores. All Rs, Rhos (*ρ* and *p* values are summarized in the Supplementary Materials.

### 4.9. Principal Component Analysis (PCA)

PCA was performed on expression levels from all markers after Z-score normalization in R using the base *stats* package and visualized in 3 dimensions using the “plot_ly” package. The 2 missing values for GAD67 and GPHN in groups CRS14 and CRS28, respectively, were imputed using SPSS (version 29) “impute missing data values” tool, imputing the mean value of each group for each missing data. The resultant loadings of each marker on the first 3 principal components were correlated using Pearson’s correlation with each PC.

### 4.10. Network Analysis

Co-expression analysis was used to investigate coordinated expression changes between markers as in [79] (and Supplementary Methods for details). Although this type of analysis is classically carried out on larger datasets, previous studies have used this approach for exploratory investigation on smaller datasets. Pairwise correlations and z-scores were calculated in SPSS version 27, and all subsequent analyses were performed in R [80], version 3.6.0. Within each group we generated z-normalized Pearson’s correlation matrices of all markers. Markers were then hierarchically clustered based on degree of correlation, and modules were generated from the resulting dendrogram using the dynamicTreeCutting function from the “WGCNA” R package (versions 4.0-4.3) [81], with a minimum module size of three. Networks were then visualized in Cytoscape version 3.9.1 [82]. Co-expression modules were compared across groups, in a pairwise manner, calculating the proportion of shared genes. The significance of observed differences was calculated using Monte Carlo based permutation testing (n = 10,000) to generate empirical *p* values. Significant *p*-values represented less preservation (i.e., the null hypothesis was that modules were identical). Benjamini Hochberg FDR correction was used to correct for multiple comparisons, after which Fisher’s *p*-value meta analysis was used to generate a single statistic for each group-to-group comparison [83].

### 4.11. Compartment Analysis

To examine group differences in the importance of each marker within compartments and each compartment within the network, we performed a compartmental hubness analysis by computing each marker’s average Kleinberg’s centrality [67] (or hubscore; see Supplementary Methods for details). Briefly, after the hierarchical clustering analysis, we extracted the regional network measures of degree, connection strength, and hubscore from the observed and permuted network data and computed empirical *p*-values. Significant *p*-values indicated a change in marker or compartment hubscore (i.e., a change in Kleinberg’s centrality). Stouffer’s *p*-value meta-analysis was used to combine marker-specific *p*-values into a single *p*-value for each marker group (synaptic compartment: GPHN, PSD95, VGLUT, and SYN1; GABAergic compartment: PV, VIP, SST, and GAD67; astroglial compartment: GLT1, GFAP, and GS) and detect potential differences in compartment-wide hubscore between the control group and the CRS groups.

## Data Availability

The raw data supporting the findings and conclusions of this article will be made available by the corresponding author (T.D.P) on request. Custom scripts and analytical code generated for this article are available at GitHub (https://github.com/jaimeknoch/banasr-lab-coexpression-compartment-analysis (accessed on 11 December 2025)) and archived on Zenodo (DOI: 10.5281/zenodo.17981401).

## Supplementary Materials

The following supporting information can be downloaded at: https://www.mdpi.com/article/10.3390/ijms27010167/s1.

## Abbreviations

BCA	Bicinchoninic Cid
cDNA	Complementary DNA
CRS	Chronic Restraint Stress
FZ	Food Zone
GABA	Gamma-aminobutyric Acid
GAD67	glutamic acid decarboxylase-67
GAPDH	glyceraldehyde 3-phosphate dehydrogenase
GFAP	glial fibrillary acidic protein
GLT1	glutamate transporter-1
GPHN	gephyrin
GS	glutamine synthase
MDD	Major Depressive Disorder
PCA	Principal Component Analysis
PFC	prefrontal cortex
PSD95	post synaptic density protein 95
PV	parvalbumin
RA	Residual Avoidance
SST	somatostatin
SYN1	synapsin-1
SZ	Shelter Zone
UCMS	Unpredictable Chronic Mild Stress
VGLUT1	vesicular glutamate transporter-1
VIP	vasoactive intestinal peptide
WGCNA	Weighted Gene Co-expression Network Analysis
