# Supplementary material for: Dynamic Behavioral and Molecular Changes Induced by Chronic Restraint Stress Exposure in Mice
Source: Int J Mol Sci. 2025 Dec 23;27(1):167. doi: 10.3390/ijms27010167 (PMC12785425; doi:10.3390/ijms27010167)
Supplement: Supplementary file 1 [file ijms-27-00167-s001.zip › ijms-3961807-supplementary.pdf]

Dynamic behavioral and molecular changes induced by chronic stress exposure in mice: importance of astroglial integrity

Thomas D. Prevot[1,2], Jaime Knoch[1,3], Dipashree Chatterjee[1,3], Sierra Codeluppi-Arrowsmith[1,3], Keith A. Misquitta[1,3], Corey J.E. Fee[1,3], Dwight Newton[1,3], Hyunjung Oh[1], Etienne Sibille[1,2,3] and Mounira Banasr[1,2,3]*

[1]Campbell Family Mental Health Research Institute of CAMH, Toronto, Canada

[2]Department of Psychiatry, University of Toronto, Toronto, Canada

[3]Department of Pharmacology and Toxicology, University of Toronto, Toronto, Canada

*Corresponding author

Mounira Banasr, Ph.D., CAMH, 250 College street, room 131, Toronto, ON M5T 1R8, Canada

Tel: 416-535-8501, ext ; E-mail: mounira.banasr@camh.ca

--- Supplementary Information ---

Table of Contents

Supplementary Materials and Methods 4
Residual Avoidance Calculation 4
qPCR 4
Network Analysis 4
Compartment Analysis 5
-Supplementary Results- 6
Behavioral assessments 6
Weekly Weight Gain 6
Weekly Coat State Score 6
Weekly testing for sucrose consumption 6
Weekly testing in the PhenoTyper Test 7
Correlation analyses between Marker Expression Levels and Behavioral Outcomes 9
Correlation Analyses Between Marker Expression Levels 11
Network Analysis. 13
- Supplementary Tables - 15

- Supplementary Figures -

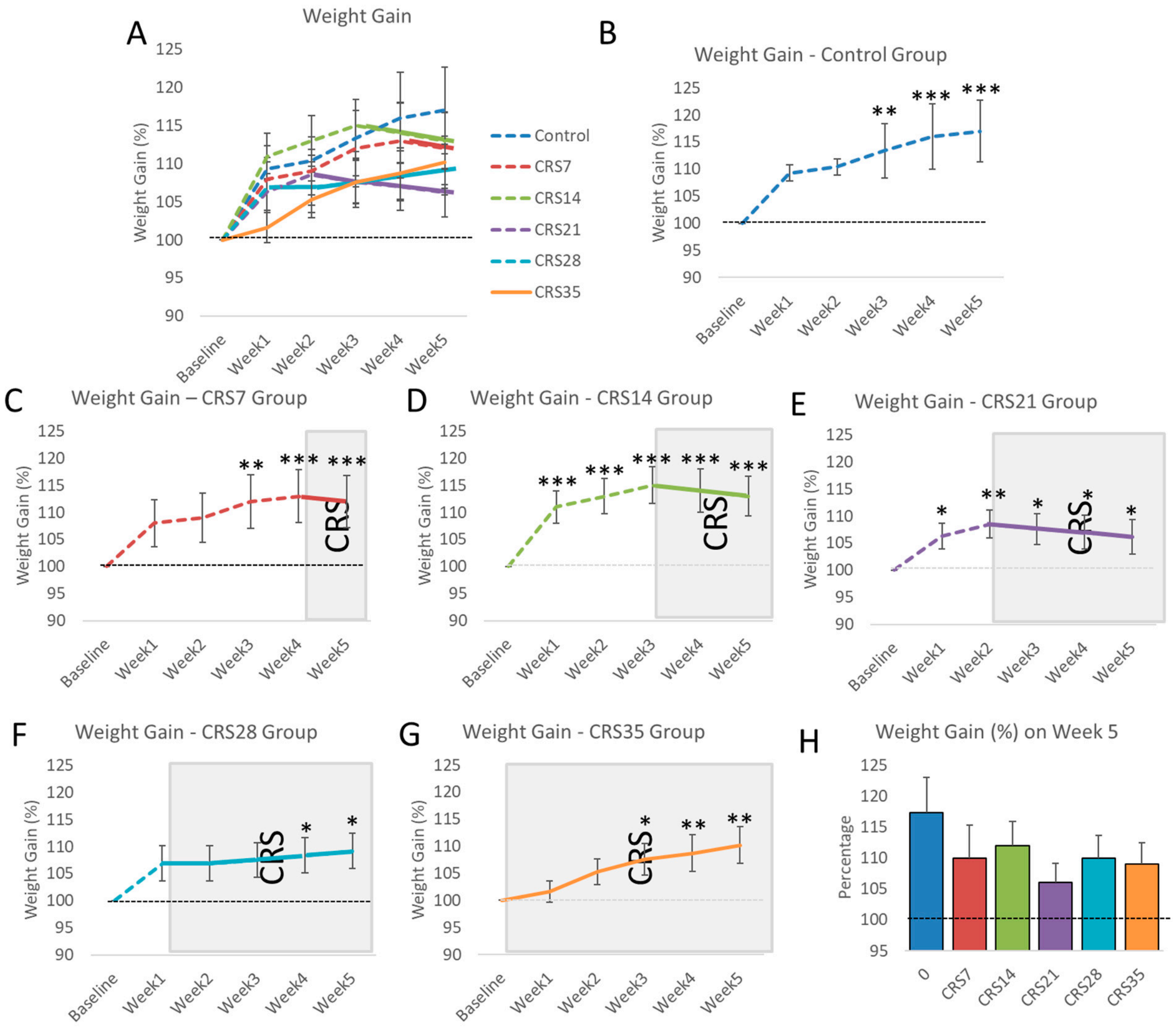

.... 23

Supplementary Materials and Methods

Residual Avoidance Calculation

We calculated the Residual Avoidance (RA) of time spent in the food zone (FZ) and shelter zone (SZ) during the PhenoTyper test as a proxy for anxiety-like/depressive-like behavior. This measure had been previously validated in our lab and peer reviewed in the paper Prevot et al., 2019. This metric represents how animals react after the light challenge as compared to controls. The RA metric can be expressed as:

$$FZRA = 100\left(1 - \frac{T_b - T_a}{\text{mean}(\hat{T}_b - \hat{T}_a)}\right)$$

Or

$$SZRA = 100\left(\frac{T_b - T_a}{\text{mean}(\hat{T}_b - \hat{T}_a)} - 1\right)$$

Where T_b is the time spent in the zone (either FZ or SZ) from 12am-5am, T_a is the time spent in the zone from 11pm-12am and ^ over top signifies the control group. If the mouse avoids the illuminated zone by either avoiding the food zone or spending the time hidden in the shelter, then RA>0. Average of the RA from the Control animals is always 0. This RA calculation informs us about the reaction after the mice after light challenge as compared to controls. RA scores were calculated in a sex-dependent manner, with the average of the RA from the male and female control groups, both being equal to 0.

qPCR

These are the primers we used from Integrated DNA Technologies (IDT; Iowa, USA):

- Actin (F: 5'-CCTAGCACCATGAAGATCAA-3'; R: 5'-GGAAGGTGGACAGTGAGG-3')
- SST (F: 5'-CAACTCGAACCCAGCAAT-3'; R: 5'-GGTCTGGCTAGGACAACAA-3')
- VIP (F: 5'-GACATCTTGCAGAATCCCTTA-3'; R: 5'-CTGCTGTAATCGCTGGTG-3')
- PVALB (PrimeTime® qPCR Primers, Mm.PT.58.7596729, Pvalb Exon Location 3-4, 20X [F+R])
- CYCLO (PrimeTime® qPCR Primers, Mm.PT.39a.2.gs, Ppia Exon Location 4-5, 20X [F+R]).
- GAPDH (PrimeTime® qPCR Primers, Mm.PT.39a.1, Gapdh Exon Location 2-3, 20X [F+R])

Network Analysis

Co-expression analysis was used to examine changes in coordinated expression between the markers in this study as in [2]. All analyses were performed in R [3], version 3.6.0. Analytical code is available upon request. Briefly, within each group we generated Pearson correlation matrices of all markers, Z-

normalized to account for marker scaling differences. Markers were then hierarchically clustered based on degree of correlation, and modules were generated from the resulting dendrogram using the dynamicTreeCutting function from the WGCNA R package [4], with a minimum module size of three. Networks were visualized in Cytoscape [5].

Co-expression modules were compared across stress groups using permutation testing to assess the degree to which they were preserved over the course of CRS. We examined the difference in module composition in each CRS group versus controls, and between each progressive CRS duration. We used a Monte-Carlo permutation approach (n=10,000) to compare observed cross tabulation-based measures of module preservation (i.e. whether markers remained co-expressed or not) to empirical resampling-based distributions of variability in module composition. These distributions were used to generate module-wise empirical p-values. For each group-wise comparison (e.g. CRS7 vs CRS0), all pairwise module comparisons were performed. Significant p-values represented a lesser degree of preservation (i.e. co-expression modules showed a different composition). Fisher's p-value meta-analysis was used to combine module-wise p-values into a single p-value for each group-to-group comparison after Benjamini-Hochberg FDR correction [6].

Compartment Analysis

In order to examine the role that chronic stress plays in changing of the GABAergic, synaptic, and astroglial compartments with exposure to chronic stress, we performed a compartmental hubness analysis by computing the average Kleinberg's centrality [7](henceforth known ashubscore, a measure eigen) of the markers within each compartment. After the hierarchical clustering analysis, using the igraph package we extracted the regional network measures of degree, connection strength, and hub score from the observed and permuted network data (n=10,000). This allowed for the calculation of hub score differences from CRS 0 for each marker and each other CRS group (e.g.,GLT1hubscore difference between CRS 0 and CRS 7). Empirical p-values were calculated for each marker by comparing the observed hub score differences to the null distribution of hub score differences generated by the permutations. Significant p-values indicate a change in marker hub score(i.e., a change in authority centrality of a specific marker in the network).Then, Stouffer's p-value meta-analysis was used to combine marker-specific p-values into a single p-value for each marker group (GABAergic compartment: PV,VIP, SST, GAD67;synapticcompartment: GPHN, PSD95, VGLUT,SYN1; astroglial compartment: GLT1, GFAP, GS)and detect potential differences between the CRS 0 group and the CRS groups.

-Supplementary Results-

Behavioral assessments

Results obtained for the between-groups cross-sectional analysis of the behavioral readouts at week 5 is in the main text. In this section of the supplemental results, we describe the results obtained every week in the **Supplementary figures S1-3**.

While including multiple timepoints corresponding to non-stress periods, these figures are included to show consistency in repeated assessments (weight gain, coat state, and phenotyper), to document baseline stability and provide internal replication of main findings once CRS exposure had begun.

Weekly Weight Gain

Repeated measures ANOVA on the weight gain measurement, assessed weekly (**Supplementary Fig.S1A**) showed no significant difference between group ($F_{(5;450)}$=0.69; p=0.62), but showed a significant effect of weeks ($F_{(5,450)}$=37.81, p<0.0001), characterized by significant increase of weight from week to week, and no interaction better factors. To better visualize this effect, weight gain was split per group (**Supplementary Fig.S1B-G**). In all groups, the effect of the weeks was confirmed (p<0.004). In control mice, significant increase in weight gain compared to baseline was found after 3 weeks and onward. Similar profile was found in the CRS7 and CRS35 groups. In the CRS14 and CRS21 groups, weight gain was significantly higher than baseline from week 1 and onward. Finally, in the CRS28 group, weight gain was significantly higher than baseline only in week 4 and week 5. ANOVA performed on the weight gain from Week 5, across groups (**Supplementary Fig.S1H**) did not reveal significant effect of CRS Duration ($F_{(5,82)}$=0.64, p=0.66), sex ($F_{(1,82)}$=0.052, p=0.82) or interaction ($F_{(5,82)}$=0.51, p=0.76).

Weekly Coat State Score

Mixed-effects model on the coat state scores over the weeks (**Supplementary Fig.S2**) showed a significant effect of group ($F_{(2.924,\ 271.9)}$=62.42, p<0.0001). This main effect of group is characterized by increasing coat state scores with increasing CRS Duration.

Weekly testing for sucrose consumption

Sucrose consumption, compared to water consumption was assessed weekly for all mice. Repeated measure ANOVA on the results obtained weekly for all groups did not show any effect

of group ($F_{(5,405)}$=1.458; p=0.21), sex ($F_{(1,405)}$=3.58, p=0.06), no group * sex interaction ($F_{(5,405)}$=0.7; p=0.56), and no group * sex * weeks interaction ($F_{(25,405)}$=0.79; p=0.75).

Weekly testing in the PhenoTyper Test

Although all mice underwent weekly PhenoTyper testing, the full sample size (94 animals) exceeded the capacity for simultaneous assessment. To accommodate this, the study was conducted in two sub-cohorts, each comprising half of the animals from every experimental group and balanced for sex. Both sub-cohorts yielded comparable outcomes. Here, we illustrate the results from one of these 2 sub-cohorts (specifically the cohort for which there was no missing animals in any group; i.e. 8 animals per group (50% females)) to report the data and statistical findings without redundancy.

As an example, mice from the CRS7 group were tested in the Phenotyper test every week, but were only subjected to stress from Week 4 to Week 5, meaning that from baseline to Week 4, they were technically similar to Control mice. **Supplementary Figure S3** shows data from 50% of all groups in the study (as a representation of the data). It shows the time spent in the Shelter zone of the Phenotyper, in all mice from baseline to Week 4 (Week 5 being presented in **Figure S1D**). Repeated measures ANOVA performed on the time spent in the shelter at baseline, showed no main effect of group ($F_{(5,504)}$=0.1, p=0.99). A main effect of time was found ($F_{(12,504)}$=66.92, p<0.001), and is characterized by increased time spent in the shelter when the light is ON from 11pm to 12am, and then increasing from 3am to 7am (as demonstrated in Prevot et al. 2019). After determination of baseline, animals from the CRS35 group started to be subjected to CRS. On Week 1, only the CRS35 group was subjected to CRS, and at this stage, for only 1 week, while the other groups remained as controls (**Supplementary Figure S3B**). Repeated measures ANOVA on the time spent in the shelter on Week 1 showed for the first time a main effect of group ($F_{(5,504)}$=3.6, p=0.008), a significant effect of time ($F_{(12,504)}$=86.06, p<0.001) and an interaction between factors ($F_{(60,504)}$=1.54, p=0.007). *Post hoc* analyses mainly showed that animals from the CRS35 group spent significantly more time in the shelter compared to control at 2am. On Week 2, animals from the CRS35 group achieved 2 weeks of CRS, while animals from the CRS28 group achieved their first week of CRS. Similarly, repeated measures ANOVA on the time spent in the shelter showed a significant effect of group ($F_{(5,504)}$=2.45, p=0.049), a significant effect of time

($F_{(12,504)}$=92.33, p<0.001) and an interaction between factors ($F_{(60,504)}$=2.04, p=0.007). *Post hoc* analyses on the time spent in the shelter at each time point showed that the CRS28 group spent significantly more time in the shelter compared to control (p=0.009). Similarly, on Week 3, mice from the CRS35, CRS28 and CRS21 groups had been subjected to 3, 2 and 1 week of CRS, respectively. Repeated measures ANOVA on the time spent in the shelter showed a significant effect of group ($F_{(5,504)}$=9.05, p<0.001), a significant effect of time ($F_{(12,504)}$=66.05, p<0.001) and an interaction between factors ($F_{(60,504)}$=3.17, p=0.007). *Post hoc* analyses revealed significant increase in time spent in the shelter, compared to control mice, at 2am and 3 am for mice of the CRS35, CRS28 and CRS21 groups (p<0.05). Finally, on Week 4, mice from the CRS35, CRS28, CRS21 and CRS14 groups were subjected to 4, 3, 2 and 1 week of CRS respectively. Repeated measures ANOVA on the time spent in the shelter showed a significant effect of group ($F_{(5,504)}$=4.16, p=0.0037), a significant effect of time ($F_{(12,504)}$=87.72, p<0.001) and an interaction between factors ($F_{(60,504)}$=2.53, p=0.007). *Post hoc* analyses revealed significant increase in time spent in the shelter, compared to control mice, at 2am for mice of the CRS35, CRS28, CRS21 and CRS14 groups (p<0.05). Also, there was a significant increase in time spent in the shelter, compared to control mice, at 3am for mice of the CRS21 and CRS14 groups (p<0.05). These trajectory analysis of the impact of CRS on a weekly basis illustrate how the Phenotyper test captures the impact, and further demonstrates its validity as shown in Prevot et al. 2019 [8].

Additionally, residual avoidance was calculated in the SZ. RA-SZ was significantly affected by group (F(5;82)=10.23; p<0.0001) and sex (F(1,82)= 14.55; p<0.001; **Fig. 1E**), with significant increase in SZ-RA in all CRS groups compared to control (p<0.05-p<0.001). The effect of sex was further characterized, and showed that males from the CRS14, CRS28 and CRS 35 groups exhibited an overall higher RA-SZ than females (**Supplement Fig. S4A**). similar approach was used in the Food Zone (FZ; **Supplementary Fig. S4B-C**). There was a significant effect of CRS duration (p<0.001) with significant increased in FZ-RA in the CRS7, CRS14, CRS28 and CRS35 groups. There was also an effect of sex (p<0.05) which was due to higher RA-FZ being higher in males than females in the CRS28 and CRS35 groups.

Correlation analyses between Marker Expression Levels and Behavioral Outcomes

Pearson's regression analysis showed no significant link overall between GFAP protein levels and weight gain, SZ or FZ residual avoidance ($|R|<0.01$, $p>0.54$). However, similar analyses performed separately in males and females showed that GFAP protein levels and weight gain or SZ residual avoidance negatively correlated in males ($|R|>0.12$, $p<0.008$) but not in the females ($|R|<0.1$, $p>0.45$) (**Supplementary Table S3**). In addition, we found a significant positive correlation between GFAP protein levels and sucrose preference (R=0., $p<0.05$; **Fig.S3A**). Although significant when including both sexes, this correlation was only maintained in males (R=0.5, $p<0.001$) and not in females (R=0.2, p=0.16). Spearman's regression analysis showed no significant correlation between GFAP protein levels and coat state score (Rho=-0.01, p=0.88), even when splitting by sex ($p>0.26$). Pearson's regression analysis between GS protein expression levels and SZ RA or sucrose consumption (**Fig.S3B**) showed a trend level ($|R|>0.19$; ps=0.07). When split by sex, regression between GS expression level and SZ RA or sucrose consumption were found significant in males ($p<0.01$ for both), but not in females. Looking at correlation between GLT1 expression levels and sucrose consumption, Pearson's regression analysis found a positive correlation (R=0.4, $p<0.001$; **Fig.S3C**), which was confirmed separately in males (R=0.4, p=0.003) and females (R=0.4, p=0.006). Focusing on males, Pearson's regression analyses found significant correlations between GLT1 expression level and weight gain (R=0.29, p=0.04), SZ RA (R=-0.5, p=0.003), and a trend with FZ RA (R=-0.24, p=0.09). Spearman's regression analysis also identified a trend towards significance between GLT1 expression level and coat state, in male mice only (Rho=-0.25, p=0.08).

Pearson's regression analyses were performed between Syn1 expression levels and behavioral outcomes, identifying significant correlations with FZ RA (R=-0.23, p=0.02), SZ RA (R=-0.23, p=0.02) and sucrose consumption (R=0.26, p=0.008; **Fig.3D-F**). When splitting by sex, all three correlations were only conserved in males ($|R|>0.3$, $p<0.03$). In addition, Spearman's regression analyses performed between Syn1 expression level and coat state scores found an overall trend towards significance (Rho=-0.23, p=0.09), which did not survive splitting by sex ($p>0.5$). Pearson's correlation analyses between vGLUT1 expression level and behavioral outcomes did not find any significant correlations ($p>0.1$), even when splitting by sex. However, Spearman's correlation analyses between vGLUT1 coat state score was significant (Rho=-0.34; p=0.02), which is

maintained even after splitting the dataset by sex (male: Rho=-0.34, p=0.02; female: Rho=-0.27, p=0.058 (trend level)). Pearson's regression analyses performed between PSD95 expression and behavioral outcome did not identify significant correlations (|R|<0.17, p>0.11). Splitting by sex, PSD95 expression levels in male mice correlated positively with sucrose consumption (R=0.32, p=0.02).

Spearman's regression analyses on GAD67 expression levels identified negative correlation with coat state (Rho=-0.4, p<0.001), which remained significant after splitting the dataset by sex (male: R=-0.38, p=0.01; female: R=-0.4, p=0.007). In males, Pearson's regression analysis found a trend between GAD67 expression levels and sucrose consumption (R=0.25, p=0.09). Looking at GPHN expression levels, Pearson's regression analyses found significant correlation with FZ RA (R=0.2, p=0.05), and SZ RA (R=0.3, p=0.002, **Fig.3G-H**). Splitting by sex, correlation with SZ RA were trending in males and females (|R|>0.2, p<0.09).

Spearman's regression analyses between GPHN expression level and coat state scores found a significant correlation (Rho=-0.22, p=0.03), which remained significant in females (Rho=-0.5, p=0.017) but not in males after splitting by sex. Pearson's regression analyses on SST expression level only showed a trend towards positive correlation with sucrose consumption (R=0.17, p=0.09), which was maintained in males (p=0.07) after splitting by sex. Focusing on male mice, SST expression levels also correlated with weight gain (R=0.35, p=0.01) and with SZ RA (R=-0.33, p=0.026). PV expression levels did not correlate with any behavioral outcome, even after splitting by sex. Pearson's regression analyses on VIP expression level only showed a significant correlation with weight gain (R=0.24, p=0.018) which was maintained as a trend in females after splitting by sex (R=0.28, p=0.057).

Finally, possible correlations between all markers and z-scores were investigated (Supplementary Figure S8). Z-score correlated with GLT1 (R=-0.3, p=0.004), vGLUT1 (R=-0.27, p=0.008), Syn1 (R=-0.32, p=0.001), GAD67 (R=-0.3, p=0.002) and was trending towards significance with PSD95 (R=-0.18, p=0.08). After splitting by sex, different profiles emerged for males and females. In males, significant correlation were found with GLT1 (R=-0.4, p=0.0004), GFAP (R=-0.29, p=0.04), Syn1 (R=-0.36, p=0.001), GAD67 (R=-0.3, p=0.01) and was trending towards significance with vGLUT1 (R=-0.26, p=0.07) and PSD95 (R=-0.18, p=0.06). In females, only vGLUT1 was significantly

correlated with the z-score (R=-0.4, p=0.005), while GPHN was trending towards significance (R=-0.25, p=0.08).

Correlation Analyses Between Marker Expression Levels

See Supplementary Table S4 for FDR-corrected q values. Pearson's regression analyses using all 11 markers found strong correlations between the three astrocytic markers (GFAP, GS, GLT1) (**Supplementary Table S4 and Supplementary Fig.S7**). The structural astroglial marker GFAP strongly correlated with the two functional astroglial markers (GS (p<0.001) and GLT1 (p<0.001), **Supplementary Fig.S7A and B**, respectively). GS and GLT1 expression levels also correlated strongly with each other (p<0.001; **Supplementary Fig.S7C**). GFAP expression levels also correlated significantly with GAD 67 (R=0.27, p=0.008), SST (R=0.28, p=0.005) and PV (R=0.33, p=0.002) expression levels. Interestingly, none of the astroglial markers correlated with the pre-synaptic markers (Syn1 and vGLUT1). However, GS correlated with the post synaptic markers PSD95 (R=0.27, p=0.009) and GPHN (-0.23, p=0.02). GS also correlated with GAD67 (R=0.24, p=0.017), and was trending with PV (r=0.17, p=0.09) and VIP (R=0.19, p=0.057). GLT1 correlated with SST (R=0.21, p=0.03) and was trending with VIP (R=0.2, p=0.06).

Regarding the synaptic markers (PSD95, Syn1, vGLUT1), they did not significantly correlate with each other, but there was a trend toward a positive correlation between PSD95 and Syn1 levels (R=0.18, p=0.08, **Supplementary Fig.S7D**). vGLUT1 correlated with GPHN (R=0.39, p<0.001, **Supplementary Fig.S7E**) and was trending with GAD67 (R=0.18, p=0.07). PSD95 correlated with GAD67 (R=0.32, p=0.001, **Supplementary Fig.S7F**) and VIP (R=0.24, p=0.02) and was trending with GPHN (R=0.18, p=0.078).

Finally, GABAergic markers significantly correlated with each other. VIP expression levels correlated with GPHN (R=0.23, p=0.02, **Supplementary Fig.S7G**), SST (R=0.27, p=0.006, **Supplementary Fig.S7H**) and PV (R=0.48, p<0.001). PV was correlated with SST (R=0.21, p=0.04, **Supplementary Fig.S7I**), and was trending with GPHN (R=0.17, p=0.088).

Splitting the dataset by sex, we can see that correlations between markers is sex-dependent (**Supplementary Table S4**). Interestingly, correlations between all glial markers were maintained,

even after splitting the dataset by sex. While no significant correlations were found between GFAP and synaptic markers, splitting by sex showed that GFAP correlated negatively with vGLUT1 (r=-0.3, p=0.03), positively with PSD95 (r=0.33, p=0.02) and was trending toward positive correlation with Syn1 (r=0.26, p=0.07) in male mice. However, in female, only vGLUT1 correlated with GFAP (r=0.3, p=0.02), but positively, i.e. in the opposite direction than male mice. Also, GFAP expression in male did not correlate with GABA markers. Only trends were observed with GAD67 (r=0.25, p=0.08) and GPHN (r=-0.26, p=0.07). In females, GFAP expression correlated with GAD67 (r=0.35 p=0.01) and SST (r=0.3, p=0.03). In male mice, other glial markers like GS and GLT1 both correlated negatively with GPHN expression (p<0.03), but not in female mice. In female mice, GS and GLT1 both correlated positively with VIP expression (p<0.047), and GLT1 also correlated positively with SST expression (p=0.01).

While synaptic markers did not correlated between each other considering the entire data set, splitting by sex showed a sex-dependent effect. Indeed, in male mice, Syn1 expression level correlated positively with PSD95 expression level (r=0.5, p<0.0001). In female mice, Syn1 expression level correlated positively with vGLUT1 expression level (r=0.34, p=0.016). In both male and female mice, vGLUT1 expression levels correlated positively with GPHN expression levels (p<0.02), confirming the results observed in full dataset. In male mice, PSD95 expression levels correlated positively with GAD67 (p=0.002) while this correlation was only trending in female mice (p=0.06). In female mice, PSD95 positively correlated with VIP expression level (p=0.04).

Finally, GABAergic markers did not correlate between each other in male mice. However, in female mice, GPHN correlated positively with SST (p=0.048), PV (p=0.001) and VIP (p=0.03). In female mice, VIP also strongly correlated positively with SST (p=0.001) and PV (p<0.001).

Altogether, analyses of the correlation between markers shows that expression levels between compartments are linked and suggests that there is a sex-dependent relationship between markers.

Network Analysis.

PCA and network co-expression analyses were used to interpret the broad patterns of marker expression in the data across durations of stress. First, we performed a simple pairwise correlation analysis, which showed that most markers were positively correlated with each other, with the astroglial markers being particularly strongly correlated with each other. (**Figure 5A**). After this, correlation matrices were Z-normalized to account for marker scaling differences. Markers were then hierarchically clustered based on degree of correlation, and modules were generated from the resulting dendrogram using the dynamicTreeCutting function from the "WGCNA" R package [64], with a minimum module size of three. Networks were visualized in Cytoscape [65]. Co-expression modules were compared across groups to assess the degree to which they were preserved between groups. We examined the difference in module composition in each CRS group versus controls, and between each CRS groups using permutation testing (n=10,000). This allowed us to compare cross tabulation-based measures of module preservation (i.e., whether markers remained co-expressed or not) and generate distributions of variability in module composition. These distributions were used to generate module-wise empirical p-values. Significant p-values represented less preservation (i.e., co-expression modules that showed a different composition). Fisher's p-value meta-analysis was used to combine module-wise p-values into a single p-value for each group-to-group comparison after Benjamini-Hochberg FDR correction [66].

To examine group differences in the relative importance of each marker within compartments and each compartment as a whole within the network, we performed a compartmental hubness analysis by computing each marker's average Kleinberg's centrality [67]. Briefly, after the hierarchical clustering analysis, we extracted the regional network measures of degree, connection strength, and hubscore from the observed and permuted network data. This allowed for the calculation of hubscore differences from controls for each marker and for each CRS group. Empirical p-values were calculated for each marker by comparing the observed hubscore differences to the null distribution of hubscore differences generated by the permutations. Significant p-values indicated a change in marker hubscore (i.e., a change in Kleinberg's

centrality). Then, Stouffer's p-value meta-analysis was used to combine marker-specific p-values into a single p-value for each marker group (synaptic compartment: GPHN, PSD95, VGLUT, SYN1; GABAergic compartment: PV, VIP, SST, GAD67; astroglial compartment: GLT1, GFAP, GS) and detect potential differences in compartment-wide hubscore between the control group and the CRS groups.

- Supplementary Tables -

Supplementary Table S1: ***List of antibodies for western blot analysis*.**

List of primary (1°) antibodies, blocking solutions, and the concentrations used for the western blot analysis for both 1° and secondary (2°) antibodies.

	Antibody	***Blocking***	***Dilution***	***Reference***
1°	Rabbit α-GLT1 (EAAT2)	5% Milk	1:2,000	Santa Cruz Biotechnology sc-15317 (Mississauga, Ontario, Canada)
	Rabbit α-GFAP	5% Milk	1:5,000	Cell Signaling Technology 12389 (Danvers, MA, USA)
	Rabbit α-GS	5% Milk	1:5,000	Abcam ab176562 (Toronto, Ontario, Canada)
	Rabbit α-Syn1	5% Milk	1:100,000	Millipore AB1543 (Etobicoke, Ontario, Canada)
	Guinea Pig α-vGLUT1	5% Milk	1:5,000	Synaptic Systems 135304 (Goettingen, Germany)
	Rabbit α-PSD95	5% Milk	1:5,000	Cell Signaling Technology 2507S (Danvers, MA, USA)
	Mouse α-GAD67	5% Milk	1:10,000	Advanced ImmunoChemical Inc (Long Beach, CA, USA)
	Rabbit α-GPHN	5% Milk	1:2,000	Cell Signaling Technology 14304S
2°	Goat α-Rabbit HRP	-	1:10,000	Vector Labs PI-1000 (Burlingame, CA, USA)
	Goat α-GuineaPig HRP	-	1:10,000	Invitrogen A18769 (Massachusetts, USA)
	Horse α-Mouse HRP	-	1:10,000	VectorLabs (Burlingame, CA, USA)

Supplementary Table S2: Dunnet's Significance Table - Shelter Zone

Compared to Control Group

Time	Group	Mean Diff.	Crit. Diff.	Sig.	Time	Group	Mean Diff.	Crit. Diff.	Sig.
7pm	7days	275.613	409.06	-	2am	7days	**1652.672**	**727.194**	**S**
	14days	163.363	409.06	-		14days	**1368.297**	**727.194**	**S**
	21days	59.196	409.06	-		21days	**1303.974**	**727.194**	**S**
	28days	72.18	409.06	-		28days	**1278.004**	**727.194**	**S**
	35days	178.453	409.06	-		35days	**1349.912**	**727.194**	**S**
8pm	7days	-101.78	269.94	-	3am	7days	**1034.479**	**723.19**	**S**
	14days	50.722	269.94	-		14days	**1002.071**	**723.19**	**S**
	21days	-81.785	269.94	-		21days	719.197	723.19	-
	28days	-56.42	269.94	-		28days	**776.283**	**723.19**	**S**
	35days	-175.809	269.94	-		35days	626.517	723.19	-
9pm	7days	-192.781	337.881	-	4am	7days	225.972	729.196	-
	14days	103.057	337.881	-		14days	88.53	729.196	-
	21days	-74.529	337.881	-		21days	224.285	729.196	-
	28days	79.942	337.881	-		28days	245.587	729.196	-
	35days	134.551	337.881	-		35days	341.691	729.196	-
10pm	7days	34.747	485.157	-	5am	7days	-237.347	789.694	-
	14days	54.225	485.157	-		14days	-119.252	789.694	-
	21days	-74.892	485.157	-		21days	-137.274	789.694	-
	28days	154.638	485.157	-		28days	-182.487	789.694	-
	35days	304.029	485.157	-		35days	-133.342	789.694	-
11pm	7days	-226.955	730.303	-	6am	7days	769.661	802.318	-
	14days	-323.581	730.303	-		14days	456.198	802.318	-
	21days	180.987	730.303	-		21days	365.311	802.318	-
	28days	-40.753	730.303	-		28days	81.416	802.318	-
	35days	105.798	730.303	-		35days	429.171	802.318	-
12am	7days	635.326	803.599	-	7am	7days	55.699	678.939	-
	14days	598.739	803.599	-		14days	675.164	678.939	-

	21days	561.795	803.599	-	21days	562.121	678.939	-
	28days	671.15	803.599	-	28days	449.868	678.939	-
	35days	**1209.212**	**803.599**	**S**	35days	577.592	678.939	-
	7days	**1129.234**	**875.213**	**S**				
	14days	**1278.286**	**875.213**	**S**				
1am	21days	727.444	875.213	-				
	28days	802.71	875.213	-				
	35days	**989.869**	**875.213**	**S**				

S=Significant

Supplementary Table S3: Marker x Behavior Correlation Table

Pearson's Regression — OVERALL

OVERALL		Weight Gain p	Weight Gain R	RA FZ p	RA FZ R	RA Shelter p	RA Shelter R	Sucrose p	Sucrose R	Z-Score p	Z-Score R
GLIA	GLT1	0.31	(0.1)	0.74	(-0.18)	0.57	(-0.22)	0.0001	(0.4)	0.004	(-0.3)
	GFAP	0.37	(-0.09)	0.81	(-0.02)	0.54	(-0.06)	0.003	(0.3)	0.13	(-0.15)
	GS	0.62	(-0.05)	0.89	(0.01)	0.07	(-0.19)	0.07	(0.19)	0.19	(-0.13)
Synaptic	vGLUT	0.18	(0.13)	0.2	(-0.1)	0.85	(0.03)	0.83	(0.05)	0.008	(-0.27)
	Syn1	0.42	(0.08)	0.02	(-0.23)	0.02	(-0.23)	0.008	(0.26)	0.0015	(-0.32)
	PSD95	0.87	(0.01)	0.61	(-0.05)	0.23	(-0.12)	0.11	(0.17)	0.08	(-0.18)
GABA	GAD67	0.42	(0.08)	0.28	(-0.1)	0.43	(-0.12)	0.48	(0.07)	0.0026	(-0.3)
	GPHN	0.35	(0.1)	0.05	(0.2)	0.002	(0.3)	0.93	(0.01)	0.65	(-0.04)
	SST	0.13	(-0.15)	0.41	(-0.02)	0.25	(-0.1)	0.09	(0.17)	0.39	(-0.09)
	PV	0.6	(0.05)	0.98	(0.00)	0.8	(-0.02)	0.91	(0.00)	0.71	(-0.04)
	VIP	0.018	(0.24)	0.81	(0.02)	0.78	(0.03)	0.49	(0.06)	0.79	(0.02)

Spearman's Regression — OVERALL

OVERALL		Coat State p	Coat State Rho
GLIA	GLT1	0.39	(-0.09)
	GFAP	0.88	(-0.01)
	GS	0.96	(0.04)
Synaptic	vGLUT	0.001	(-0.33)
	Syn1	0.099	(-0.23)
	PSD95	0.23	(-0.13)
GABA	GAD67	0.0003	(-0.4)
	GPHN	0.03	(-0.22)
	SST	0.41	(0.084)
	PV	0.61	(0.05)
	VIP	0.14	(0.15)

p< 0.1 0.05 0.010 0.0010

p (Rho)

MALE		Weight Gain p	Weight Gain R	RA FZ p	RA FZ R	RA Shelter p	RA Shelter R	Sucrose p	Sucrose R	Z-Score p	Z-Score R
GLIA	GLT1	0.04	(0.29)	0.09	(-0.24)	0.003	(-0.5)	0.003	(0.4)	0.0004	(-0.4)
	GFAP	0.008	(0.39)	0.27	(-0.16)	0.004	(-0.46)	0.0003	(0.5)	0.04	(-0.29)
	GS	0.09	(0.25)	0.33	(-0.1)	0.01	(-0.34)	0.007	(0.4)	0.07	(-0.26)
Synaptic	vGLUT	0.12	(0.23)	0.83	(-0.03)	0.9	(0.01)	0.55	(-0.09)	0.25	(-0.17)
	Syn1	0.75	(0.08)	0.01	(-0.35)	0.03	(-0.3)	0.02	(0.33)	0.01	(-0.36)
	PSD95	0.5	(0.1)	0.18	(-0.2)	0.37	(-0.13)	0.02	(0.32)	0.06	(-0.18)
GABA	GAD67	0.97	(0.004)	0.25	(-0.1)	0.12	(-0.2)	0.09	(0.25)	0.01	(-0.3)
	GPHN	0.24	(0.17)	0.16	(0.2)	0.09	(0.2)	0.68	(-0.06)	0.79	(-0.04)
	SST	0.01	(0.35)	0.39	(-0.12)	0.026	(-0.33)	0.07	(0.26)	0.25	(-0.17)
	PV	0.9	(0.02)	0.77	(-0.04)	0.29	(-0.16)	0.91	(0.02)	0.96	(-0.007)
	VIP	0.17	(0.2)	0.84	(0.03)	0.21	(0.18)	0.63	(0.07)	0.82	(0.03)

MALE		Coat State p	Coat State Rho
GLIA	GLT1	0.08	(-0.25)
	GFAP	0.63	(0.07)
	GS	0.64	(0.07)
Synaptic	vGLUT	0.02	(-0.34)
	Syn1	0.23	(-0.17)
	PSD95	0.25	(-0.17)
GABA	GAD67	0.01	(-0.38)
	GPHN	0.18	(-0.19)
	SST	0.72	(0.05)
	PV	0.93	(0.01)
	VIP	0.92	(0.01)

FEMALE		Weight Gain p	Weight Gain R	RA FZ p	RA FZ R	RA Shelter p	RA Shelter R	Sucrose p	Sucrose R	Z-Score p	Z-Score R
GLIA	GLT1	0.84	(0.03)	0.4	(-0.12)	0.85	(0.02)	0.006	(0.4)	0.45	(-0.1)
	GFAP	0.45	(0.11	0.68	(-0.06)	0.63	(0.07)	0.16	(0.2)	0.15	(0.2)
	GS	0.3	(0.15)	0.14	(0.21)	0.75	(0.04)	0.44	(-0.1)	0.65	(0.06)
Synaptic	vGLUT	0.75	(0.05)	0.16	(-0.2)	0.6	(0.08)	0.1	(0.24)	0.005	(-0.4)
	Syn1	0.33	(0.14	0.72	(-0.05)	0.69	(-0.05)	0.43	(0.11)	0.41	(-0.12)
	PSD95	0.74	(0.05)	0.77	(0.04)	0.32	(-0.14)	0.85	(0.02)	0.41	(-0.12)
GABA	GAD67	0.33	(0.14)	0.86	(-0.02)	0.88	(0.02)	0.48	(-0.1)	0.1	(-0.23)
	GPHN	0.93	(0.002)	0.62	(0.07)	0.09	(0.24)	0.12	(0.22)	0.08	(-0.25)
	SST	0.87	(0.02)	0.37	(-0.13)	0.9	(0.01)	0.46	(0.17)	0.55	(-0.09)
	PV	0.5	(0.1)	0.45	(-0.1)	0.47	(-0.1)	0.43	(0.12)	0.12	(-0.22)
	VIP	0.057	(0.28)	0.36	(-0.13)	0.22	(-0.1)	0.36	(0.13)	0.67	(0.06)

FEMALE		Coat State p	Coat State Rho
GLIA	GLT1	0.47	(0.1)
	GFAP	0.26	(-0.16)
	GS	0.99	(-0.002)
Synaptic	vGLUT	0.058	(-0.27)
	Syn1	0.72	(-0.05)
	PSD95	0.72	(-0.05)
GABA	GAD67	0.007	(-0.4)
	GPHN	0.017	(-0.5)
	SST	0.74	(0.05)
	PV	0.52	(-0.09)
	VIP	0.27	(0.16)

p< 0.1 0.05 0.010 0.0010

Supplementary Table S4: Marker x Marker Correlation Table

Overall		Glia				Synaptic								GABA							
		GS		GLT1		Syn1		vGLUT		PSD95		GPHN		GAD67		SST		PV		VIP	
		q	(r)	q	(r)	q	(r)	q	(r)	q	(r)	q	(r)	q	(r)	q	(r)	q	(r)	q	(r)
GLIA	GFAP	1.00E-04	(0.62)	1.00E-04	(0.55)	0.740	(-0.03)	0.800	(0.02)	0.055	(0.19)	0.670	(0.04)	0.008	(0.27)	0.005	(0.28)	0.002	(0.33)	0.110	(0.16)
	GS			1.00E-04	(0.49)	0.130	(0.16)	0.400	(-0.08)	0.009	(0.27)	0.020	(-0.23)	0.017	(0.24)	0.880	(0.01)	0.090	(0.17)	0.057	(0.19)
	GLT1					0.120	(0.16)	0.160	(0.14)	0.250	(0.12)	0.310	(-0.1)	0.920	(0.01)	0.030	(0.21)	0.290	(0.1)	0.062	(0.2)
Synaptic	Syn1							0.190	(0.13)	0.080	(0.18)	0.950	(0.006)	0.380	(0.09)	0.950	(0.005)	0.350	(-0.09	0.410	(0.08)
	vGLUT									0.710	(-0.03)	1.00E-04	(0.39)	0.070	(0.18)	0.630	(-0.05)	0.200	(-0.13)	0.490	(-0.07)
	PSD95											0.078	(0.18)	0.001	(0.32)	0.880	(0.01)	0.290	(0.1)	0.016	(0.24)
	GPHN													0.190	(0.13)	0.350	(0.09)	0.088	(0.17)	0.020	(0.23)
GABA	GAD67															0.220	(-0.12)	0.160	(0.14)	0.650	(-0.04)
	SST																	0.040	(0.21)	0.006	(0.27)
	PV																			1.00E-04	(0.48)
	VIP																				

q (R) p< 0.1 0.05 0.010 0.0010 Underlined value survived FDR correction

♂		Glia				Synaptic								GABA							
		GS		GLT1		Syn1		vGLUT		PSD95		GPHN		GAD67		SST		PV		VIP	
		q	(r)	q	(r)	q	(r)	q	(r)	q	(r)	q	(r)	q	(r)	q	(r)	q	(r)	q	(r)
GLIA	GFAP	1.00E-04	(0.5)	1.00E-04	(0.6)	0.070	(0.26)	0.030	(-0.3)	0.020	(0.33)	0.070	(-0.26)	0.080	(0.25)	0.200	(0.19)	0.110	(0.23)	0.820	(-0.03)
	GS			1.00E-04	(0.61)	0.025	(0.33)	0.120	(0.22)	0.105	(0.24)	0.020	(-0.32)	0.150	(0.21)	0.590	(-0.08)	0.110	(0.23)	0.450	(0.11)
	GLT1					0.230	(0.18)	0.350	(0.13)	0.880	(0.02)	0.030	(-0.32)	0.920	(0.01)	0.770	(0.04)	0.910	(-0.017)	0.290	(-0.15)
Synaptic	Syn1							0.660	(-0.06)	4.00E-04	(0.5)	0.850	(0.02)	0.140	(0.22)	0.540	(0.09)	0.120	(-0.23)	0.540	(0.09)
	vGLUT									0.750	(-0.04)	4.00E-04	(0.5)	0.080	(0.25)	0.290	(-0.15)	0.054	(-0.28)	0.330	(-0.14)
	PSD95											0.070	(0.26)	0.002	(0.45)	0.270	(-0.16)	0.300	(-0.15)	0.340	(0.14)
	GPHN													0.420	(0.12)	0.500	(-0.1)	0.100	(-0.24)	0.480	(0.1)
GABA	GAD67															0.420	(-0.12)	0.250	(0.17)	0.460	(-0.11)
	SST																	0.430	(0.11)	0.450	(0.11)
	PV																			0.300	(0.15)
	VIP																				

♀		Glia				Synaptic								GABA							
		GS		GLT1		Syn1		vGLUT		PSD95		GPHN		GAD67		SST		PV		VIP	
		q	(r)	q	(r)	q	(r)	q	(r)	q	(r)	q	(r)	q	(r)	q	(r)	q	(r)	q	(r)
GLIA	GFAP	1.00E-04	(0.8)	1.00E-04	(0.6)	0.460	(-0.1)	0.020	(0.3)	0.430	(0.11)	0.110	(0.22)	0.010	(0.35)	0.030	(0.3)	0.060	(0.26)	0.190	(0.19)
	GS			0.004	(0.4)	0.630	(-0.06)	0.670	(0.06)	0.038	(0.3)	0.640	(-0.06)	0.060	(0.26)	0.340	(0.13)	0.210	(0.18)	0.047	(0.28)
	GLT1					0.370	(0.13)	0.011	(0.36)	0.220	(0.17)	0.400	(0.12)	0.970	(0.004)	0.010	(0.36)	0.160	(0.2)	0.011	(0.36)
Synaptic	Syn1							0.016	(0.34)	0.740	(0.05)	0.170	(0.2)	0.640	(-0.07)	0.880	(0.02)	0.270	(0.16)	0.160	(0.2)
	vGLUT									0.830	(-0.03)	0.020	(0.31)	0.350	(0.13)	0.620	(0.07)	0.890	(-0.02)	0.870	(-0.02)
	PSD95											0.470	(0.1)	0.064	(0.27)	0.300	(0.15)	0.090	(0.24)	0.041	(0.3)
	GPHN													0.090	(0.24)	0.048	(0.28)	0.001	(0.45)	0.030	(0.3)
GABA	GAD67															0.490	(-0.1)	0.160	(0.2)	0.950	(0.009)
	SST																	0.120	(0.22)	0.001	(0.48)
	PV																			1.00E-05	(0.58)
	VIP																				

Supplementary Table S5: Principal Component Analysis

Total Variance Explained

Component	Initial Eigenvalues			Extraction Sums of Squared Loadings		
	Total	% of Variance	Cumulative %	Total	% of Variance	Cumulative %
1	2.692	24.477	24.477	2.692	24.477	24.477
2	1.612	14.654	39.131	1.612	14.654	39.131
3	1.547	14.060	53.191	1.547	14.060	53.191
4	1.208	10.978	64.169	1.208	10.978	64.169
5	1.079	9.809	73.978	1.079	9.809	73.978
6	.758	6.892	80.871			
7	.681	6.186	87.057			
8	.458	4.166	91.223			
9	.412	3.750	94.973			
10	.337	3.068	98.041			
11	.216	1.959	100.000			

Extraction Method: Principal Component Analysis.

Correlation with Principal Components (r

Marker	PC1	PC2	PC3
**GAD67**	0.353	0.238	0.545
**SST**	0.362	0.088	0.507
**PV**	0.523	0.252	0.484
**VIP**	0.532	0.322	0.479
**GLT1**	0.658	0.294	0.113
**GFAP**	0.803	0.183	0.058
**GS**	0.731	0.431	0.234
**GPHN**	0.116	0.866	0.005
**vGLUT**	0.026	0.513	0.484
**Syn1**	0.184	0.055	0.397
**PSD95**	0.47	0.24	0.257

Supplementary Table S6. Significant and Trending Changes in Hubscore: Males

a- Hubscores per marker

CRS Duration	Group	Marker	Direction of Change from CRS 0	P value
**CRS 7**	GABAergic	PV	↓	**0.0149**
	Synaptic	PSD95	↑	**0.0126**
	Astroglial	GLT1	↓	**<0.0001**
**CRS 14**	Astroglial	GFAP	↑	**<0.0001**
		GS	↑	**<0.0001**
**CRS 21**	GABAergic	GAD67	↑	**<0.0001**
	Synaptic	PSD95	↑	**<0.0001**
		SYN1	↓	**0.0019**
	Astroglial	GLT1	↓	**<0.0001**
**CRS 28**	GABAergic	GAD67	↑	**<0.0001**
	Synaptic	GPHN	↓	**0.003**
		VGLUT	↓	**<0.0001**
	Astroglial	GFAP	↑	**<0.0001**
		GS	↑	**<0.0001**
**CRS 35**	GABAergic	GAD67	↑	**<0.0001**
	Synaptic	PSD95	↑	**0.0032**
	Astroglial	GFAP	↑	**0.0277**
		GS	↑	**<0.0001**

All other non-reported values had p-values >0.05.

b- Average hubscore per group marker

CRS Duration	Group	Direction of Change	p-value
**CRS 7**	GABAergic	—	
	Synaptic	—	
	Astroglial	—	
**CRS 14**	GABAergic	—	
	Synaptic	—	
	Astroglial	↑	**<0.0001**
**CRS 21**	GABAergic	—	
	Synaptic	↓	**0.0004**
	Astroglial	—	
**CRS 28**	GABAergic	↑	**0.0158**
	Synaptic	—	
	Astroglial	↑	**<0.0001**
**CRS 35**	GABAergic	↑	**0.0382**
	Synaptic	—	
	Astroglial	↑	**0.0074**

Supplementary Table S7. Significant and Trending Changes in Hubscore: Females

a- Hubscores per marker

**CRS Duration**	**Group**	**Marker**	**Direction of Change from CRS 0**	**P value**
**CRS 7**	GABAergic	SST	↑	*0.061*
	Synaptic	GPHN	↓	**<0.0001**
	Astroglial	GS	↑	**<0.0001**
**CRS 14**	GABAergic	SST	↑	**<0.0001**
	Synaptic	PSD95	↑	**0.0136**
	Astroglial	GS	↑	**<0.0001**
**CRS 21**	GABAergic	PV	↓	**<0.0001**
		SST	↑	*0.0649*
	Synaptic	GPHN	↓	**<0.0001**
		VGLUT	↑	**0.0008**
**CRS 28**	GABAergic	SST	↑	**<0.0001**
		GAD67	↑	*0.0951*
	Synaptic	GPHN	↓	**0.0002**
		PSD95	↑	*0.0737*
	Astroglial	GLT1	↓	**<0.0001**
		GFAP	↓	**<0.0001**
**CRS 35**	GABAergic	SST	↑	**0.0086**
	Synaptic	VGLUT	↑	*0.0772*
	Astroglial	GLT1	↓	**<0.0001**
		GFAP	↓	**0.0025**

All other non-reported values had p-values >0.05, trending p values denoted by italics (0.1< ***p*** *< 0.05)*

b- Average hubscore per group marker

**CRS Duration**	**Group**	**Direction of Change**	**p-value**
**CRS 7**	GABAergic	—	
	Synaptic	—	
	Astroglial	↑	**0.051**
**CRS 14**	GABAergic	↑	**0.0139**
	Synaptic	—	
	Astroglial	↑	**0.016**
**CRS 21**	GABAergic	—	
	Synaptic	—	
	Astroglial	—	
**CRS 28**	GABAergic	↑	**0.0034**
	Synaptic	—	
	Astroglial	↓	**0.012**
**CRS 35**	GABAergic	—	
	Synaptic	—	
	Astroglial	↓	**0.052**

# - Supplementary Figures -

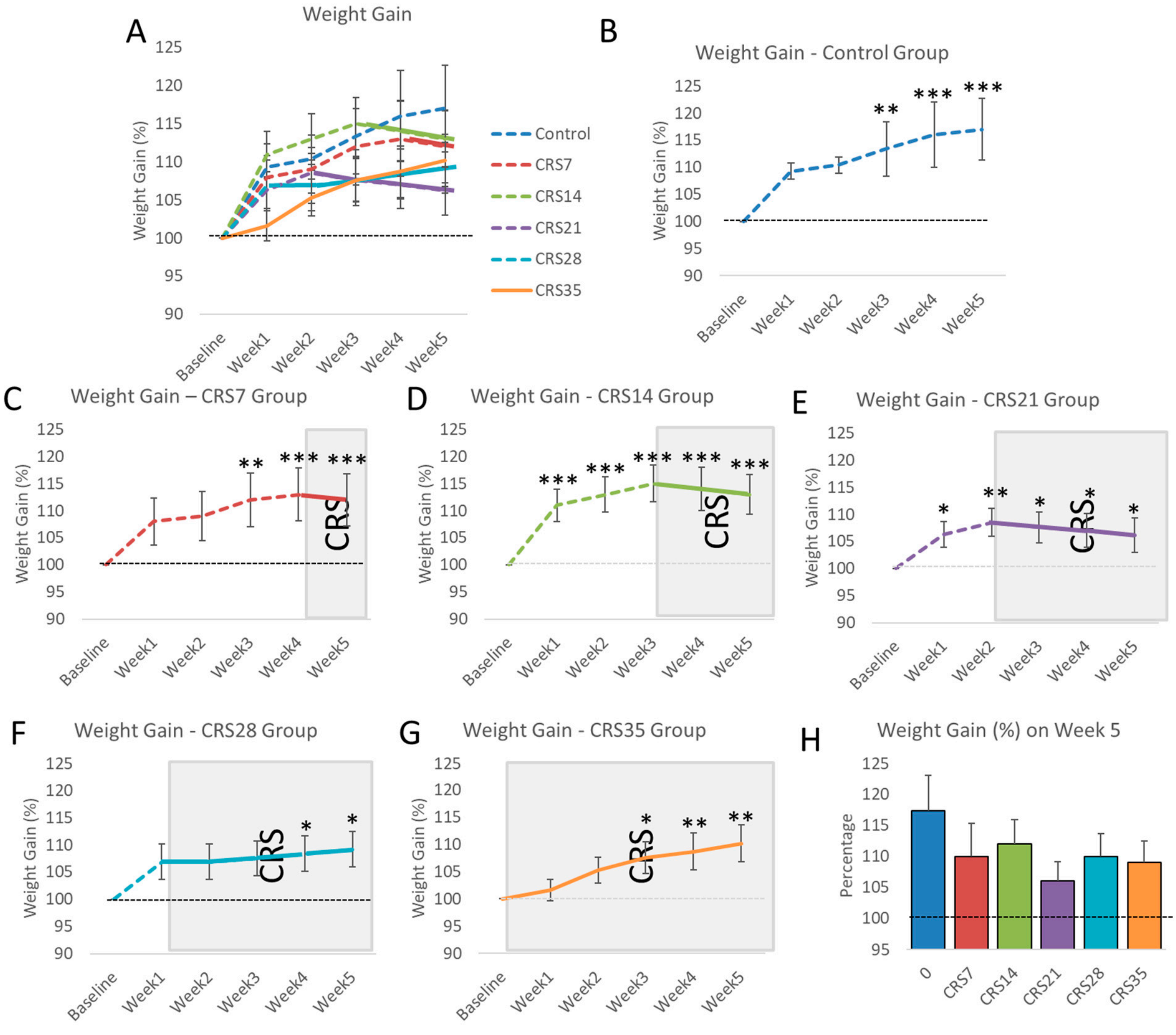

Supplementary Figure S1: Evolution of weight gain over weeks

Mice were weighted on a weekly basis over the course of the experiment. Weight gain was measured from their baseline weight (representing 100%), and was calculated every week. Overall weight gain results including all groups is presented in the panel A, and split per group in the panels B through G. The black dotted line represents 100%. The colored dotted lines represent the weight gain when the mice are not subjected to CRS. Plain lines represent the period when the are subjected to CRS, also presented by the gray zone in the background. Panel H presents the overal weight gain measured after Week 5, i.e after completion of the entire study. *p<0.05;**p<0.01, ***p<0.001 compared to Baseline.

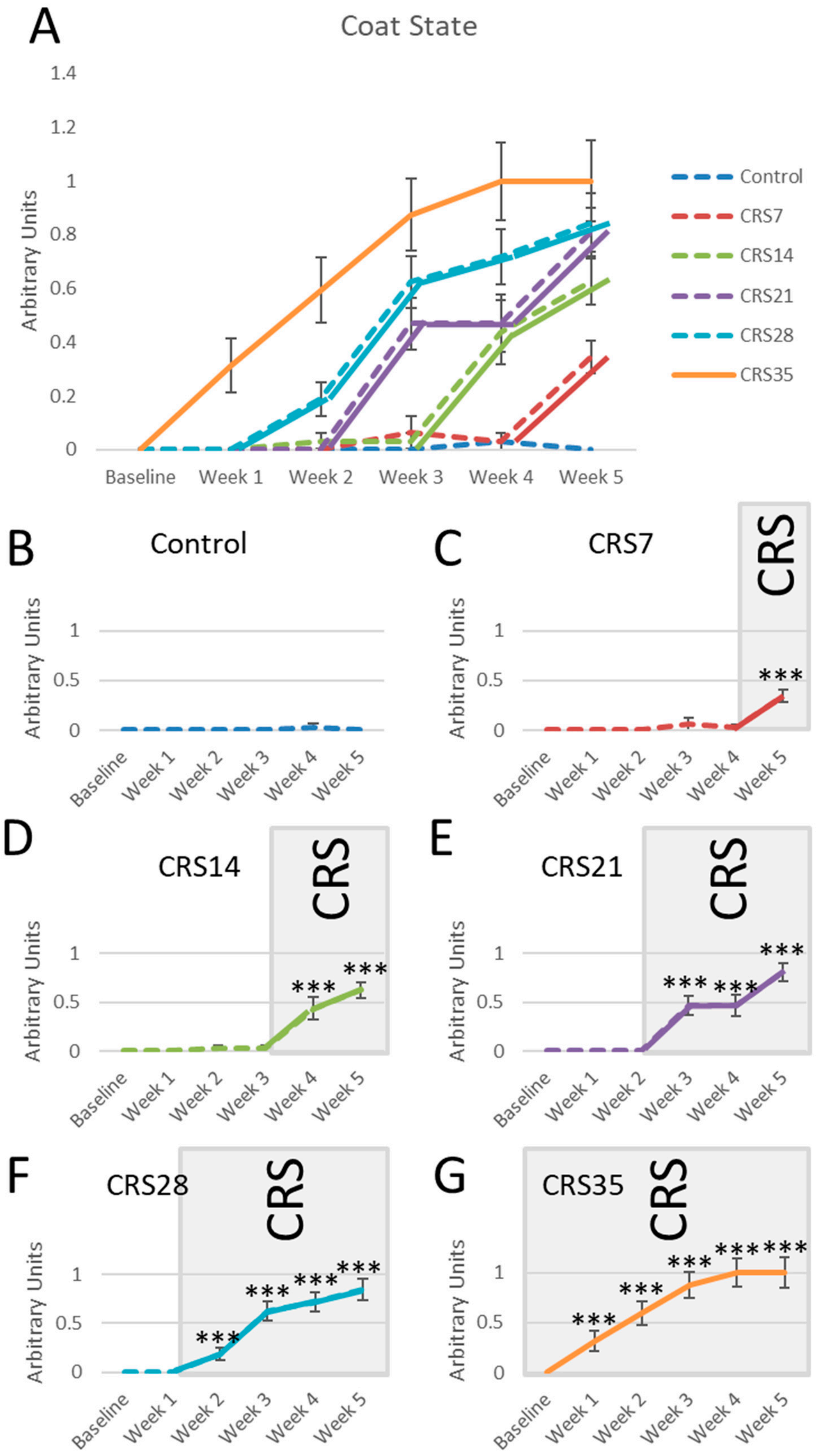
A
Coat State
Arbitrary Units
Baseline
Week 1
Week 2
Week 3
Week 4
Week 5
Control
CRS7
CRS14
CRS21
CRS28
CRS35
B
Control
C
CRS7
D
CRS14
E
CRS21
F
CRS28
G
CRS35
CRS
***

Supplementary Figure S2: Evolution of Coat State over the weeks.

Coat state was assessed every week, based on the method described in Yalcin et al (2015). Panel A provided an overview of the coat state in all groups, throughout the entire study. Panels B through G represent the different groups starting with the Control group (B) and finishing with the CRS35 group (G). The colored dotted lines represent the coat state when the mice are not subjected to CRS. Plain lines represent the period when the are subjected to CRS, also presented by the gray zone in the background. *$p<0.05$;**$p<0.01$, ***$p<0.001$ compared to Baseline.

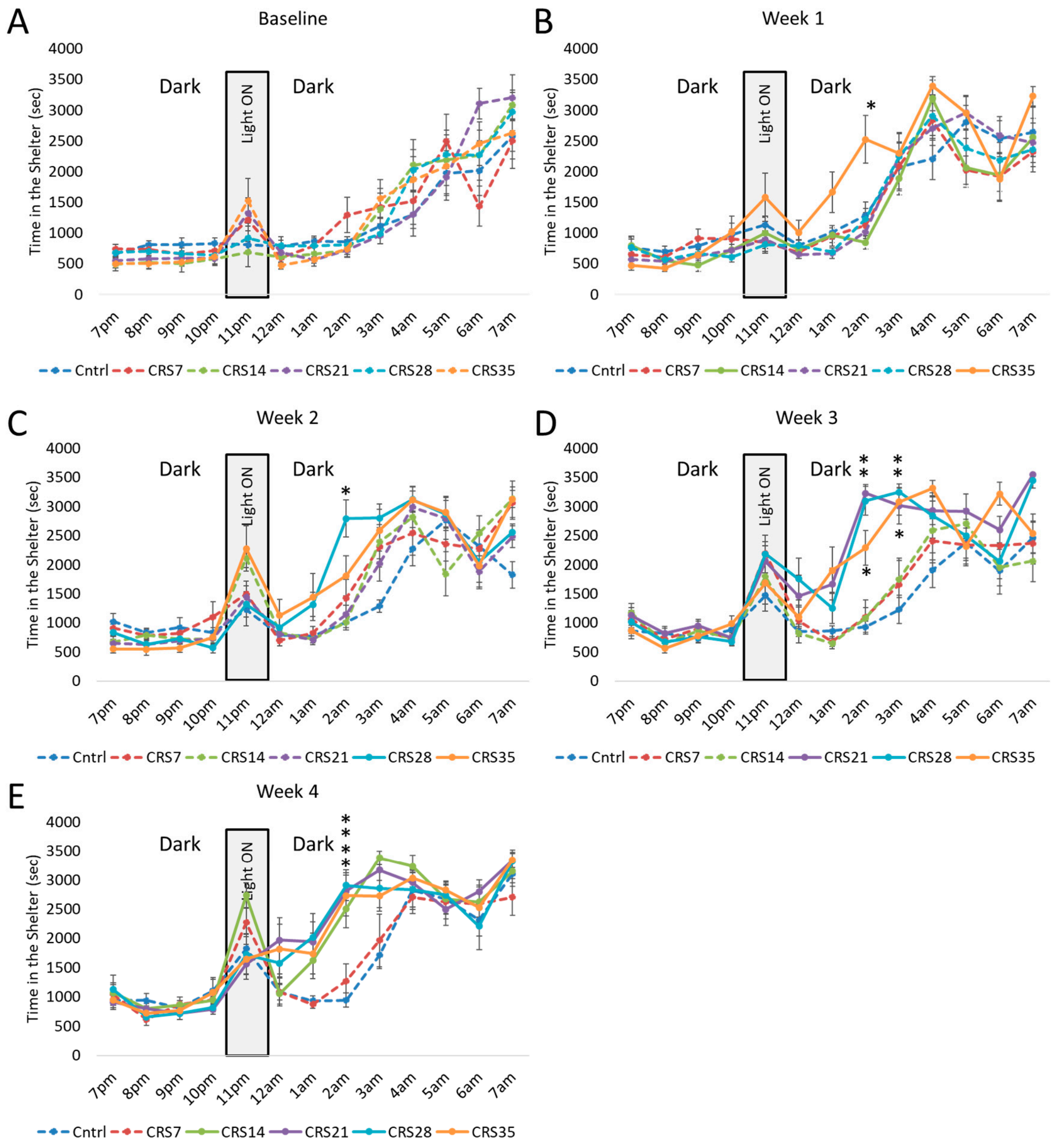

Supplementary Figure S3: Assessment of anxiety-like behaviors in the Phenotyper on a weekly basis.

Mice from all groups were tested in the Phenotyper test, whether their exposure to CRS had started or not. Before initiation of the CRS paradigm, mice were tested in the Phenotyper test for baseline activity (A), showing no difference between groups, assigned artificially at this stage. Then, on Week 1 (B), only the animals from the CRS35 group had started CRS, while the others were kept as control. On this week, we showed a significant effect of CRS, characterized by increasing time in the shelter, in particular at 2am. This is observed on Week 2 (C), 3 (D) and 4 (E). In this figure, groups subjected to CRS are represented with plain lines. Groups that were not subjected to CRS yet, are presented with dotted lines. The gray

zone in the background of each graph represents the time when the light was turned ON, as an acute challenge. Data are presented as average per group ± SEM. *$p<0.05$ compared to Control mice.

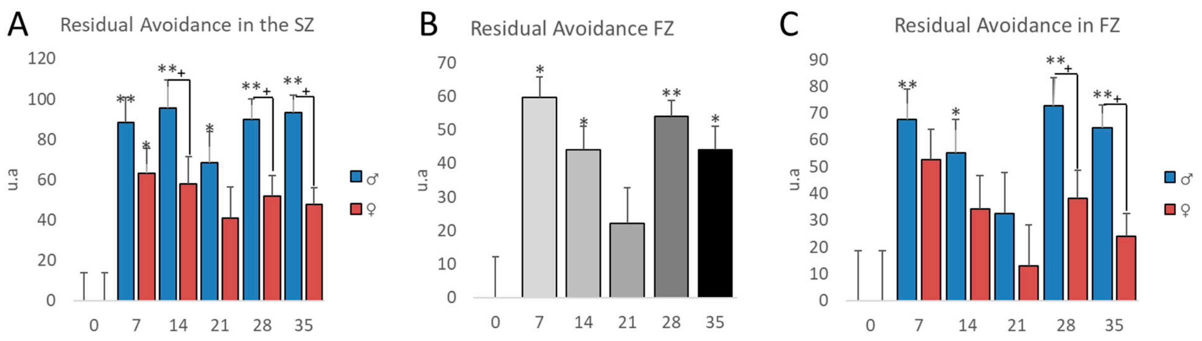

Supplementary Figure S4: Residual avoidance in the Shelter and in the Food Zones, including sex differences

Residual avoidance was calculated in a sex-dependent manner, with male and female RA from the Control group both being equal to 0. Overall effect of CRS Duration was significant, even after splitting the dataset by sex, in the Shelter Zone (SZ; Panel A). The effect of sex showed that males from the CRS14, CRS28 and CRS 35 groups exhibited an overall higher RA score than females. Residual avoidance was calculated in the Food Zone (B) and showed significant effect of the CRS Duration ($F_{(5;82)}$=6.9; p<0.001), an effect of Sex ($F_{(1;82)}$=9.5; p=0.002) and no Duration*Sex interaction (p>0.05). *Post hoc* Dunnett's test revealed an increase in RA score compared to Control after 7, 14, 28 and 35 days of CRS (p<0.05), but not after 21 days of CRS (p=0.6). In the FZ, the effect of sex was characterized and showed higher scores in males after 28 and 35 days of CRS (C). *p<0.05;**p<0.01 compared to Control; +p<0.05 compared to Females.

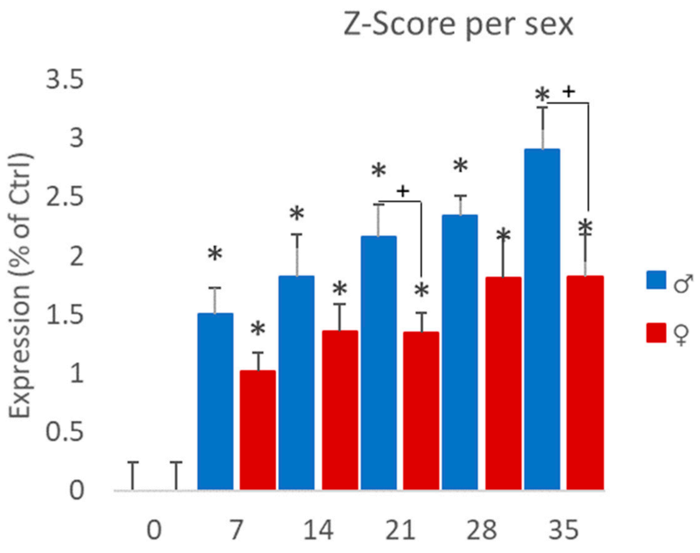

Supplementary Figure S5: Z-Score per sex.

Z-scores were calculated in a sex-dependent manner, with both z-scores from male and female control groups being equal to 0. ANOVA performed on the Z-Score showed a significant effect of CRS Duration ($F_{(5,82)}$=18.2, p<0.001), a significant effect of Sex ($F_{(1,82)}$=12.9, p=0.0006) and no CRS Duration*Sex interaction (p>0.5). *Post hoc* analyses show that in both males and females there is a significant increase of the z-score in all CRS duration groups, compared to the Control group (ps<0.01). The effect of Sex is explained by a Z-score higher in males than females. *p<0.05 compared to Control; +p<0.05 compared to Females.

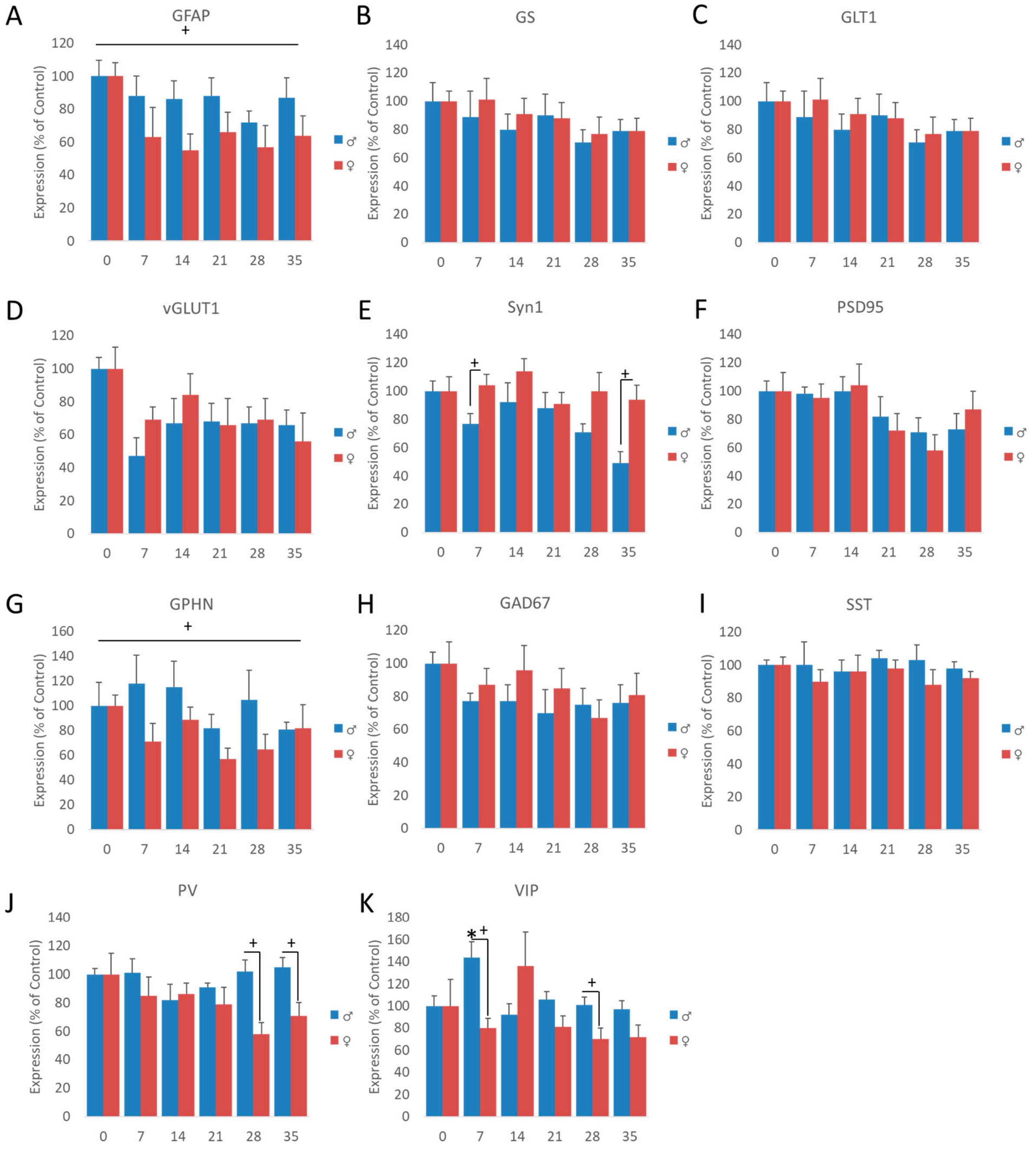

Supplementary Figure S6: Cellular markers per sex.

Expression levels of each marker was assessed in a sex-dependent manner. Statistical analyses performed on GFAP expression levels showed an overall effect of sex (A). No effects of sex were observed on expression levels of GS (B), GLT1 (C) or vGLUT1(D). Syn1 expression levels were significantly lower in males compared to females in the CRS7 and CRS35 groups (E). No effects of sex were observed on expression

levels of PSD95 (F) and GAD67 (G). An overall effect of sex was identified on GPHN expression levels (H). No effects of sex were observed on expression levels of SST (I). A significant effect of sex was identified in PV expression levels, characterized by lower expression levels in females compared to males in CRS28 and CRS35 groups (J). Finally, an interaction between CRS Duration*Sex was observed on VIP expression levels, which was characterized by higher expression levels in male compared to females in the CRS7 and CRS28 groups (K). *$p<0.05$ compared to Control; +$p<0.05$ compared to Females.

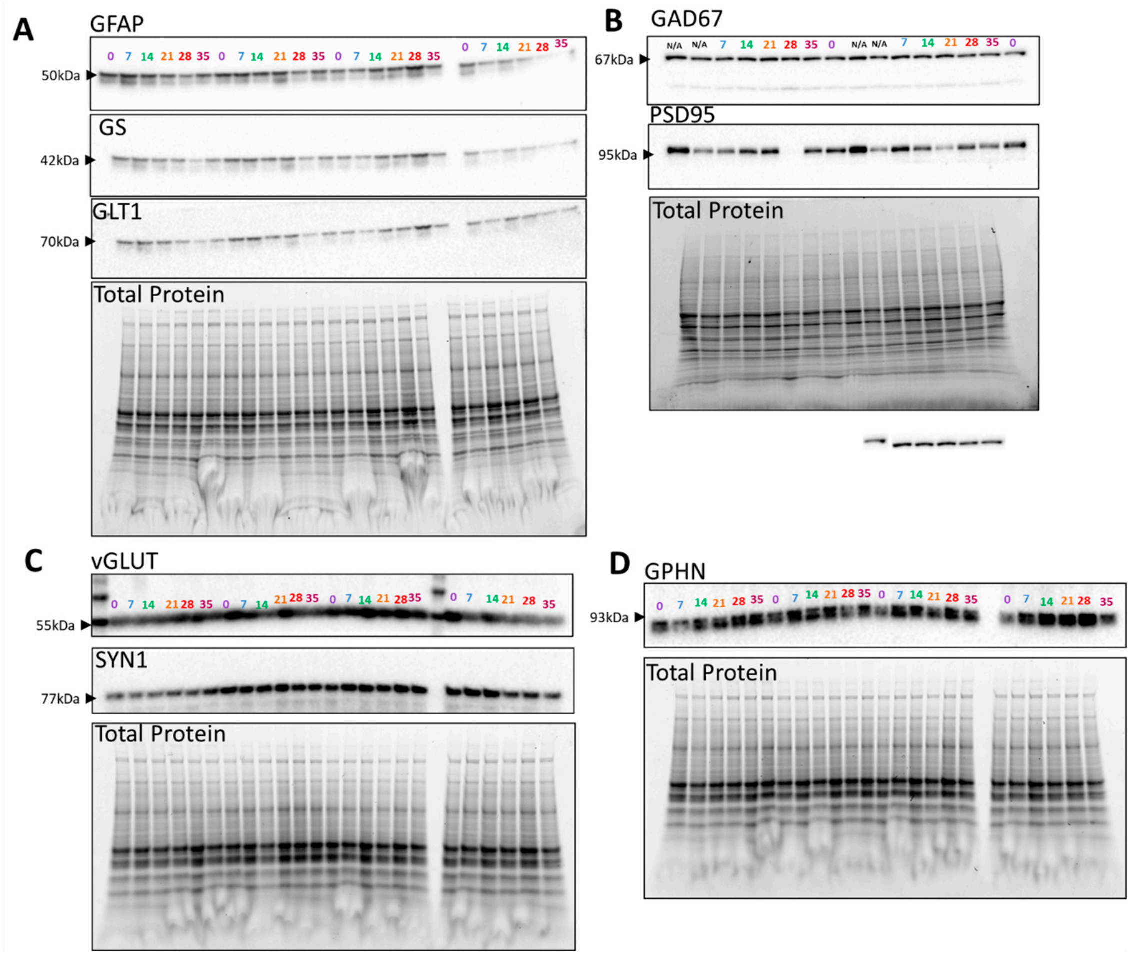

Supplementary Figure S7: Representative images of Western blots, with gel image.

Membrane images with protein were captured on a ChemiDoc. Protein presented are GFAP, GS and GLT1 from the same gel (A), GAD67 and PSD95 from another gel (B), vGLUT1 and SYN1 from another gel (C) and GPHN from another gel (D). For each, the StainFree gel image capturing the total protein is included. *Colored numbered represent the CRS duration with 0 being the control group. N/A represents samples that are not included in this publication.*

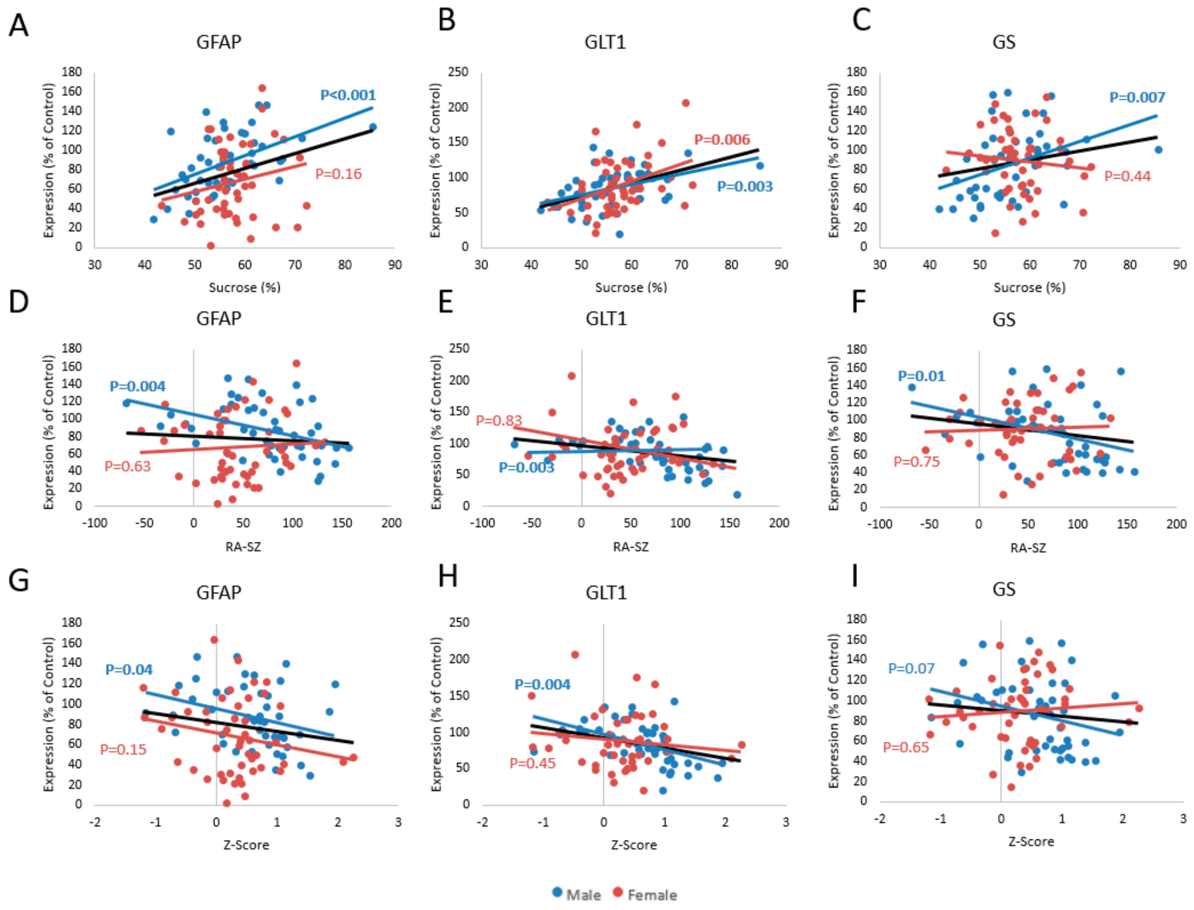

Supplementary Figure S8: Behavioral outcomes and glial marker correlations split by sex.

Pearson's regression analyses were performed between glial markers and behavioral outcomes, and split by sex. GFAP, GLT1 and GS expression levels correlated with Sucrose consumption in male but not in females (A-C). GFAP, GLT1 and GS expression levels also correlated with residual avoidance in the shelter in males but not in females (D-F). Finally, GFAP and GLT1 expression levels in males, but not in females, correlated with z-scores (G-H), while GS expression levels were only trending towards significance (I).

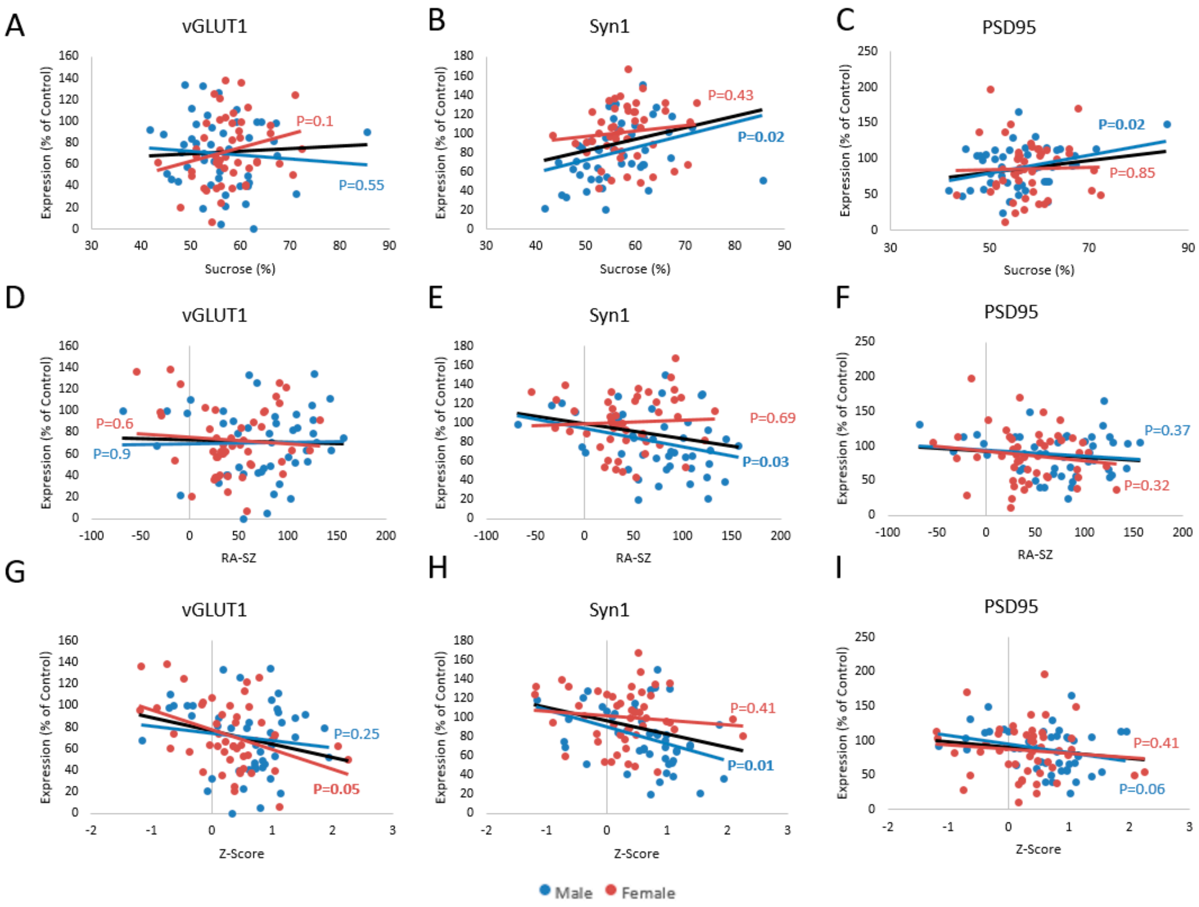

Supplementary Figure S9: Behavioral outcomes and synaptic marker correlations split by sex.

Pearson's regression analyses were performed between synaptic markers and behavioral outcomes, and split by sex. vGLUT1 expression levels did not correlate with sucrose consumption, neither in males nor in females (A). In males, Syn1 and PSD95 expression levels correlated with sucrose consumption but not in females (B-C). Regarding correlating with residual avoidance in the shelter, vGLUT and PSD95 expression levels did not correlated with RA-SZ neither in males nor in females (D and F), while Syn1 expression levels correlated with RA-SZ in males only (E). vGLUT1 expression levels correlated with z-score in females but not in males (G). Syn1 expression levels correlated with z-score only in males (H). Finally, PSD95 expression levels did not correlate with z-scores, however levels in males were trending towards significance (I).

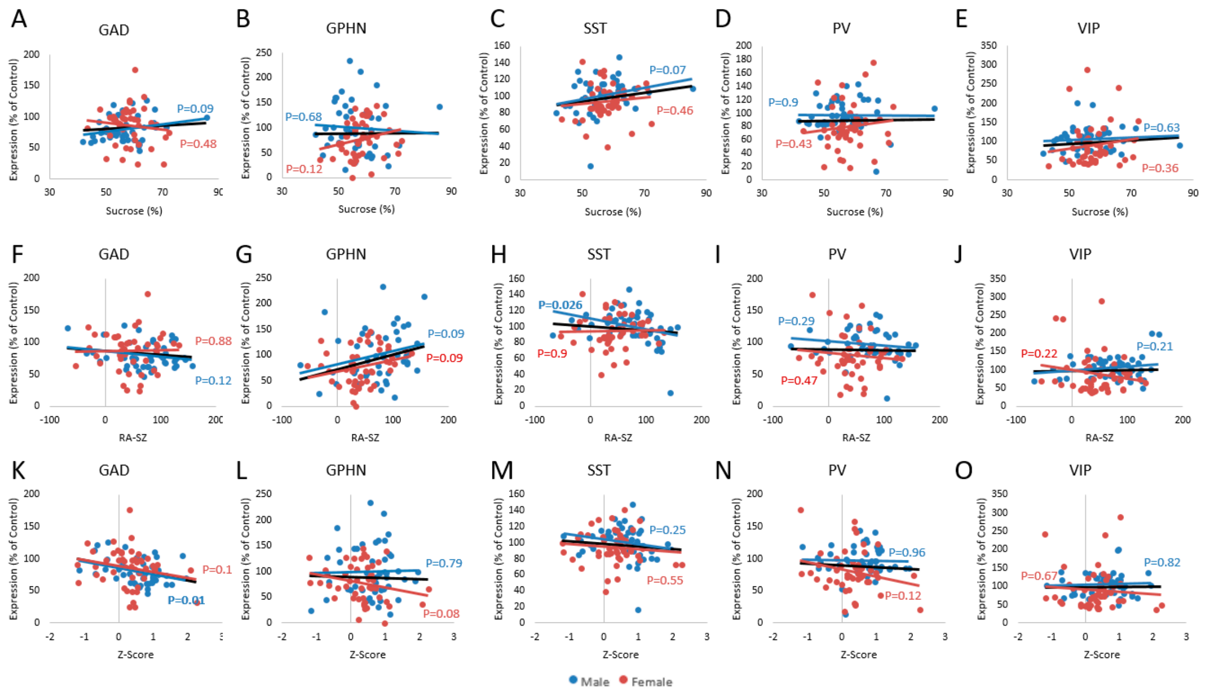

Supplementary Figure S10: Behavioral outcomes and GABAergic marker correlations split by sex.

Pearson's regression analyses were performed between GABAergic markers and behavioral outcomes, and split by sex. Sucrose consumption did not correlate with expression levels of GAD (A), GPHN (B), SST (C), PV (D) or VIP (E) neither in males nor in females. Regarding residual avoidance in the shelter (RA-SZ), it did not correlate with GAD expression levels, neither in males nor in females (F). Expression levels of GPHN were trending in both males and females (G). RA-SZ correlated negatively with SST expression levels in males but not in females (H). Regarding PV and VIP expression levels, they did not correlate with RA-SZ, neither in males nor in females (I-J). Z-scores did not correlate with GAD expression levels (K), GPHN (L), SST (M), PV (N) or VIP (O), neither in males nor in females.

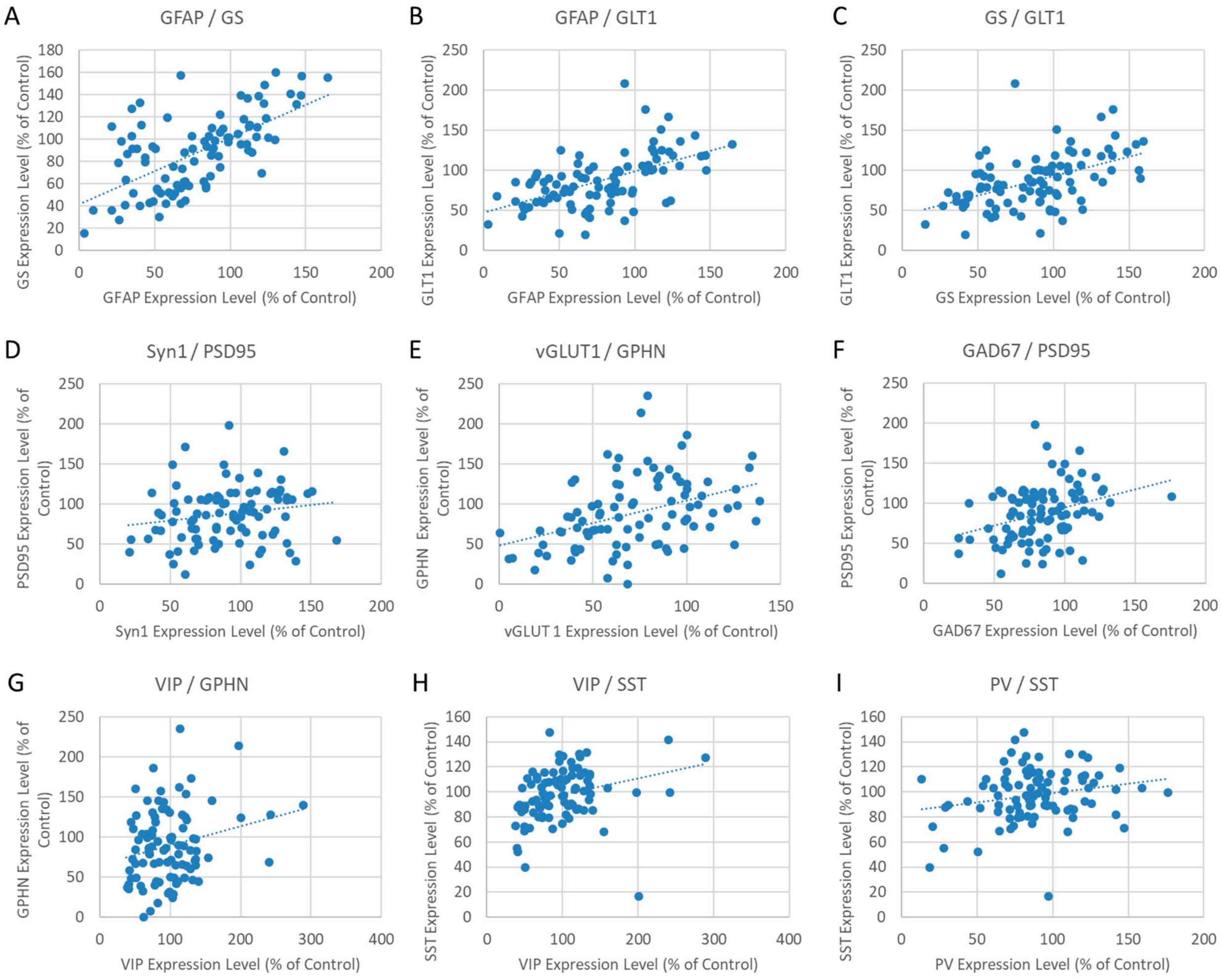

Supplementary Figure S11. Between Markers Correlations.

Pearson's regression analyses between GFAP and GS (A), and GLT1 (B) showed significant positive correlation (ps<0.001). GS and GLT1 expression levels also correlated strongly with each other (p<0.001; C). Pearson's regression analyses also showed a trend toward a positive correlation between PSD95 and Syn1 levels (R=0.18, p=0.08, D). vGLUT correlated with GPHN (p<0.001, E). PSD95 correlated with GAD67 (p=0.001,F). Finally, many of the GABAergic markers significantly correlated with each other. VIP expression levels correlated with GPHN (p=0.02, G) and SST (p=0.006, H). PV was correlated with SST (p=0.04, I).

Supplementary Figure S11: 3D scatterplots.

See special files:

- Full_Data 3D animated (sex difference) PCA plot
- Full_Data 3D animated PCA plot
- Full_Data 3D no_animation (sex difference) PCA plot
- Full_Data 3D no_animation PCA plot

REFERENCES

1. Yalcin, I., F. Aksu, and C. Belzung, *Effects of desipramine and tramadol in a chronic mild stress model in mice are altered by yohimbine but not by pindolol.* Eur J Pharmacol, 2005. **514**(2-3): p. 165-74.
2. Philip, V., et al., *Transcriptional markers of excitation-inhibition balance in germ-free mice show region-specific dysregulation and rescue after bacterial colonization.* J Psychiatr Res, 2021. **135**: p. 248-255.
3. Team, R.C., *R: A language and environment for statistical computing.* 2013.
4. Langfelder, P. and S. Horvath, *WGCNA: an R package for weighted correlation network analysis.* BMC Bioinformatics, 2008. **9**(1): p. 559.
5. Lopes, C.T., et al., *Cytoscape Web: an interactive web-based network browser.* Bioinformatics, 2010. **26**(18): p. 2347-8.
6. Heard, N.A. and P. Rubin-Delanchy, *Choosing between methods of combining p-values.* Biometrika, 2018. **105**(1): p. 239-246.
7. Kleinberg, J.M. *Authoritative sources in a hyperlinked environment*. in *SODA*. 1998.
8. Prevot, T.D., et al., *Residual avoidance: A new, consistent and repeatable readout of chronic stress-induced conflict anxiety reversible by antidepressant treatment.* Neuropharmacology, 2019. **153**: p. 98-110.
